# Unveil Intrahepatic Cholangiocarcinoma Heterogeneity through the Lens of Omics and Multi-Omics Approaches

**DOI:** 10.3390/cancers16162889

**Published:** 2024-08-20

**Authors:** Veronica Porreca, Cristina Barbagallo, Eleonora Corbella, Marco Peres, Michele Stella, Giuseppina Mignogna, Bruno Maras, Marco Ragusa, Carmine Mancone

**Affiliations:** 1 Department of Molecular Medicine, Sapienza University of Rome, 00161 Rome, Italy; corbella.2016275@studenti.uniroma1.it (E.C.); peres.1461165@studenti.uniroma1.it (M.P.); 2 Section of Biology and Genetics, Department of Biomedical and Biotechnological Sciences, University of Catania, 95123 Catania, Italy; cbarbagallo@unict.it (C.B.); michelestella7@gmail.com (M.S.); mragusa@unict.it (M.R.); 3 Department of Biochemistry Science, Sapienza University of Rome, 00185 Rome, Italy; pina.mignogna@uniroma1.it (G.M.); bruno.maras@uniroma1.it (B.M.)

**Keywords:** intrahepatic cholangiocarcinoma, tumor microenvironment, tumor heterogeneity, patients’ stratification, personalized medicine

## Abstract

**Simple Summary:**

Intrahepatic cholangiocarcinoma (iCCA) is a highly aggressive primary liver cancer leading to death in 10–15% of cases. In recent years, the iCCA worldwide incidence has been increasing, and research is currently focused on identifying valuable diagnostic biomarkers and developing specific therapies to counteract the disease despite its high heterogeneity. The use of cutting-edge and broad-spectrum applications like omics represents a successful approach to studying the intricate pathobiology of iCCA. In this review, we discuss how both single- and multi-omics studies can pave the way for identifying potential biomarkers, and more importantly, for stratifying iCCA patients. This stratification aims to enhance the therapeutic intervention through personalized medicine (PM).

**Abstract:**

Intrahepatic cholangiocarcinoma (iCCA) is recognized worldwide as the second leading cause of morbidity and mortality among primary liver cancers, showing a continuously increasing incidence rate in recent years. iCCA aggressiveness is revealed through its rapid and silent intrahepatic expansion and spread through the lymphatic system leading to late diagnosis and poor prognoses. Multi-omics studies have aggregated information derived from single-omics data, providing a more comprehensive understanding of the phenomena being studied. These approaches are gradually becoming powerful tools for investigating the intricate pathobiology of iCCA, facilitating the correlation between molecular signature and phenotypic manifestation. Consequently, preliminary stratifications of iCCA patients have been proposed according to their “omics” features opening the possibility of identifying potential biomarkers for early diagnosis and developing new therapies based on personalized medicine (PM). The focus of this review is to provide new and advanced insight into the molecular pathobiology of the iCCA, starting from single- to the latest multi-omics approaches, paving the way for translating new basic research into therapeutic practices.

## 1. Introduction

Intrahepatic cholangiocarcinoma (iCCA) is an aggressive and lethal epithelial neoplasm that arises from the malignant transformation of cholangiocytes lining bile ducts of the liver [1,2,3,4]. Initially, iCCA develops in the hepatic parenchyma as a mass injury in the absence of jaundice or other symptoms. As a result, many iCCA patients are asymptomatic and the tumor injuries are typically discovered accidentally through radiographic examinations conducted for other purposes [5]. Currently, available chemotherapy and radiotherapy for iCCA are poor and have limited effectiveness, offering only palliative benefits [6,7]. In this scenario, surgical resection is the only well-defined therapeutic option to counteract the disease [8]. Unfortunately, due to the size and the extent of the lesions in iCCA patients, only one-third of them are eligible for surgical resection [9]. The poor chances of early diagnosis and effective therapeutic approaches, including systemic treatments, result in a life expectancy of around 5 years for these patients, as reported in statistics and medical case studies [10,11].

To date, iCCA is estimated as the second most common primary liver malignancy after hepatocellular carcinoma (HCC), accounting for 10–15% of cases [12,13,14,15]. However, in recent years, this hepatobiliary cancer has revealed interest and concern due to its increased incidence in several areas of the world [14,16]. The large geographical discrepancy in iCCA prevalence, with a higher incidence in Eastern Asia than in Western countries, is related to the complex interaction between genetic predisposition and the higher prevalence of geographical risk factors [17,18]. Progressively, this geographical and epidemiological disparity is increasingly diminishing. In the last three decades, there has been reported a boost in iCCA rates in Western Europe and the United States among people aged 45–59 [9,19,20,21].

Epidemiological and experimental investigations have characterized and confirmed some of the risk factors associated with the occurrence of iCCA. Among them, the most acclaimed are viral infections (liver flukes, viral B and C hepatitis), primary sclerosing cholangitis, metabolic syndrome, smoking, alcohol abuse, and cirrhosis [22,23]. In addition, some environmental components are responsible for the geographical discrepancy in iCCA prevalence. For example, it was reported that *Opisthorchis Viverrini*, a liver fluke endemic to Southeast Asia, infects much of the population, and is considered a major etiologic agent for iCCA [2,23,24]. Unlike, in Western countries, iCCA is often associated with primary sclerosing cholangitis and other diseases associated with chronic liver inflammation such as nonalcoholic fatty liver disease (NAFLD) [17,18]. Overall, these clinical features make iCCA a tumor with marked heterogeneity, distinguishing it from the other two forms of cholangiocarcinoma, perihilar CCA (pCCA) and distal CCA (dCCA). This underscores the need for studies to develop specific guidelines for this type of bile duct cancer [25].

Tumor heterogeneity is an issue of increasing interest and attention in the field of cancer research as it affects both drug resistance and disease progression, hindering the improvement in outcomes of patients [26,27]. It is divided into inter-tumor, referring to genotypic and phenotypic differences between patients, and intratumor, based on biological and genomic variations within the tumor. The former is affected by etiological and environmental factors, the latter by the microenvironment surrounding the tumor [28,29]. In the frame of the iCCA, tumor heterogeneity is characterized by a wide spectrum of phenotypic and molecular characteristics.

iCCA has been classified into two main subtypes: Large-Duct Type (LDT) and Small-Duct Type (SDT) [30]. LDT develops in the larger intrahepatic bile ducts located in the perihilar portion of the liver and is morphologically composed of mucin cells. Conversely, SDT affects small ducts localized in the peripheral parts of the liver and is represented by non-mucin-secreting cells [31,32]. This classification underlies a high inter-tumor heterogeneity that arises from multifactorial etiologies often correlated to genomic alterations, which, in turn, are responsible for the three main patterns of growth observed in iCCA: mass forming, periductal infiltrating, and intraductal growing [14,33]. On the other hand, intratumor heterogeneity is associated with multiple molecular alterations at the genomics, epigenomics, proteomics, and metabolomics levels among cancer cells, as well as their dynamic interactions with the tumor microenvironment (TME) [34,35].

iCCA exhibits a distinctive TME characterized by excessive infiltration of immune and stromal cells [36]. The strong active crosstalk between cancer and non-cancerous cells promotes an aberrant extracellular matrix (ECM) deposition, the inhibition of the immune response, and different regulation of angiogenesis and lymphangiogenesis [37]. This results in a highly reactive desmoplastic, hypovascularized, and pro-lymphangiogenic TME, which exacerbates the malignant phenotype of the cancer cells and promotes metastatic spread through the lymphatic system [38,39,40,41].

Overall, this high inter- and intratumor heterogeneity combined with peculiar TME explains not only the resistance of the iCCA to current therapies but also the challenge in identifying biomarkers useful for early diagnosis. Additionally, it complicates the development of personalized medicine (PM) approaches, which are essential for ensuring better prognoses and outcomes for patients. Based on these premises, iCCA research shifts from the “one size fits all” paradigm and moves toward an omics approach. This direction aims to develop specific patient stratifications and to understand the molecular features of its intricate pathogenesis [42,43,44].

This review aims to examine the “omics profile” of the iCCA highlighting how the high-throughput approaches, from single to multiple methodologies, can be valuable and cutting-edge for both basic research and clinical practice. Given the high number of papers available in the literature, we decided to focus our attention on the studies published in the last decade able to address patient’s stratifications by using at least 30 tumor sample specimens.

## 2. Omics Cancer Era: From Single- to Multi-Omics Approaches

One of the main challenges of cancer research is identifying effective approaches to monitor and manage the complexity and multifactorial nature of tumors [45]. Tumor cells reprogram their cellular fate, and understanding the underlying mechanism represents the central focus of cancer studies. This altered program is characterized by apoptosis resistance, uncontrolled growth, and the acquisition of a malignant phenotype that promotes metastatic dissemination. These cellular behaviors lead to the development of intricate and heterogeneous molecular patterns within the same type of tumor and among different patients [46]. Changes in gene and transcript expression profiles subsequently lead to the alteration of the proteomics and metabolomics landscapes. Conventional methods often fall short of providing a comprehensive understanding of the complex molecular factors driving cancer initiation, progression, and metastasis. Faced with the challenge of addressing and troubleshooting the intricate network linked to tumor heterogeneity, how can we identify specific biomarkers involved in cancer pathophysiology to develop new cutting-edge therapies?

To answer this question, research has focused on developing advances and high-throughput technologies that have completely revolutionized the scientific field, particularly based on single-cell techniques.

### 2.1. Single-Based Omics

The “omics” era refers to innovative methods capable of simultaneously analyzing a single aspect of the cell life cycle on a large scale in terms of genomics, transcriptomics, proteomics, and metabolomics analyses. Defined as the “four big omics”, they play a pivotal role in enhancing our understanding of cancer pathobiology, providing a holistic perspective on the molecular events underpinning its development. Particularly, these approaches can offer a detailed characterization of the intra-cell molecular heterogeneity and identify suitable models for investigating novel mechanisms underlying specific cancer phenotypes [47].

Genomics represents the most widely studied omics field in cancer research, as it can address the cancer cells’ high genetic heterogeneity and genomic instability. It is based on sequencing of entire genomes allowing analysis of their structure, function, and evolution. Indeed, whole-genome sequencing (WGS) data derived from new applications like DNA microarray, next-generation sequencing (NGS), and the third generation of long reads sequencing (TGS) have the potential to characterize inter-individual variations, revealing cancer-associated traits like coding or non-coding driver mutations and mutational signatures, which are patterns of germline and somatic mutations [48,49,50,51,52,53]. Initially, intratumor heterogeneity was primarily attributed to genetic mutations. However, this concept is now recognized as more multifaceted, with epigenetic mechanisms also playing a significant role [54,55]. Therefore, epigenomics is considered another omics branch that can elucidate the epigenetic landscape of the cell–cell variations in terms of chromatin remodeling based on reversible DNA or histone modifications that affect gene expression without modifying the DNA sequence [56,57]. It has been demonstrated that DNA methylation plays a central role in carcinogenesis, promoting hypo- and hyper-methylation states to influence and regulate oncogenes and onco-suppressor genes [58].

In recent years, a widely used omics technique has focused on single-cell RNA sequencing (scRNA-seq), which enables the detection of the transcriptomics profile at the single-cell level, generally subjected to sudden changes arising from the development stage as well as from internal and external stimuli [59]. Since the transcriptome is highly dynamic compared to the genome, high-throughput technologies can detect these fluctuations by identifying and quantifying coding and non-coding RNA transcribed from the genome at a given time of the cell’s life cycle [60]. The preponderant role of transcriptomics in the tumor context is evidenced by data demonstrating the involvement of microRNAs (miRNAs) and long non-coding RNAs (lncRNAs) in tumor-suppressing or tumor-promoting, highlighting them as potential cancer biomarkers [61,62].

Together with genomics, proteomics is playing an increasingly major role in cancer research. It is known that many biological functions in cancer are triggered by proteins. Proteomics allows us to understand the entire sets of proteins (proteome) produced by a cell, tissue, or organism, enabling the quantification of their abundance, modifications, and interactions. This high-throughput approach can identify potential biomarkers and patterns of protein expression useful for tumor prognosis, tumor classification, and target therapies [63,64,65]. In addition, proteomics technology provides information about protein targets and signaling pathways related to uncontrolled growth and metastatic activity of cancer cells [66,67]. It is important to note that the proteome is markedly more complex than the transcriptome. While transcriptomics faces challenges due to its dependence on temporal dynamics and alternative splicing, the complexity of the proteome arises from spatiotemporal dynamics and a range of post-translational modifications (PTMs) such as proteolysis, glycosylation, phosphorylation, nitrosylation, and ubiquitination. Particularly, PTMs are involved in intracellular signaling, controlling enzymatic activities, and maintaining cell structure. In this context, advancements in tandem mass spectrometry (LC-MS/MS) techniques have enabled the identification of thousands of proteins in cell or body fluids and additionally to discriminate PTMs by defining the corresponding shift in the mass of the protein [68,69].

Concurrently, proteomics can be implemented with the development of new digital imaging and multiplexed immunohistochemistry (mIHC). With the support of the mIHC, histology has also become part of the quantitative “omics” era, allowing for a more accurate description of the tissue’s architecture, but above all for analyzing simultaneously the expression profiles of a multitude of proteins in a tissue section at a single-cell resolution. This spatial technology allows for accurately identifying cellular types and phenotypes in the context of tissue and across the patient’s cohort [70,71,72].

Finally, metabolomics is focused on the quantification of metabolites that are produced by cellular metabolic activities, particularly amino acids, carbohydrates, lipids, and nucleotides. In addition, metabolites are not only the final products of gene and protein expression but also the consequence of the mutual relationship between the genome and the internal environment [73,74]. Abnormalities in metabolite levels are found in many pathological states, including cancer [75]. In tumor pathogenesis, cancer cells undergo metabolic reprogramming that enables their survival and proliferation as observed in altered glycolytic fluxes and glucose metabolism [76]. Metabolomics is therefore becoming an essential tool in cancer biology, with a strong potential regarding the use of possible markers for diagnosis and follow-up of therapy without invasive approaches in the patients [77,78,79].

Overall, single-omics studies provide valuable insight into cancer’s molecular fingerprints, aiding in understanding the intratumor heterogeneity. However, to fully understand the cancer progression mechanisms, develop new therapeutic approaches, and identify more sensitive cancer biomarkers, omics strategies still require further refinement and enhancement.

### 2.2. Multi-Omics Approaches

Single-omics approaches can detect a vast amount of information simultaneously but they only provide insight into a part of the dynamic process of gene expression and regulation. While genomics, transcriptomics, proteomics, and metabolomics offer precise information about specific processes, they fail to present an exhaustive portrait of the genotype-to-phenotype relationship and to explain the causality from molecular alterations to the manifestation of disease. To overcome these limitations, new methodological approaches based on computational analysis methods enable the simultaneous profiling of multiple omics. This “multi-omics revolution” has the potential to transform our understanding of cell heterogeneity in health and disease. In the holistic analysis of complex biological processes, the integration of single-omics data into multi-omics studies is one of the most important challenges. This has led to the development of a wide range of bioinformatic tools and methods (i.e., Network approach, Bayesian approach, Fusion-based approaches, Similarity-based approaches, Correlation approach, and multivariate methods) to address biological questions of interest such as the disease subtyping, the prediction of biomarkers, and in bridging the gap from genotype to phenotype [80]. All these methods, by adopting mathematical integrative approaches, allow for assessing the flow of information from one omics level to the other, thus improving prognostics and predictive accuracy of disease phenotypes and paving the way for better treatments by PM. However, since these tools are largely isolated and not easy to use, the future outlook is to develop standard strategies that can minimize the technical variability to achieve well-matched multimodal data with easy and biologist-friendly visualization and interpretation.

**Figure 1:**
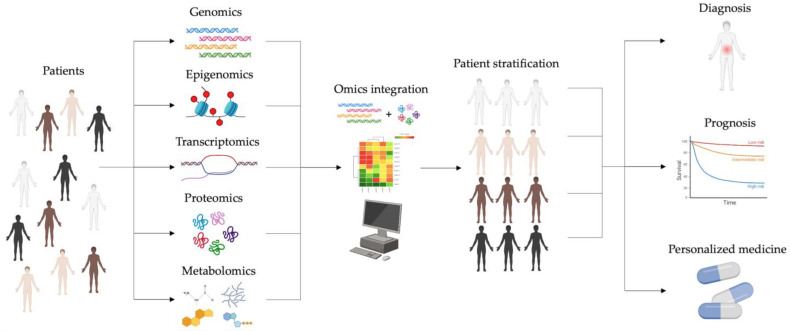
The multi-omics workflow in cancer. Single-omics datasets from different patients are integrated using bioinformatic tools to generate a patient stratification to enable personalized diagnosis, prognosis, and medical treatments. Inter-tumor heterogeneity of patients is indicated with people with different colors.

The central focus of this new vision in cancer research is to better understand both inter and intratumor heterogeneity. This complexity makes it challenging to identify highly sensitive and specific molecular targets for cancer treatment and prevention. The idea of developing a PM for cancer patients is an emerging field with the potential to adapt therapies toward cancer drivers and modulate the immune environment of the tumor [81]. To meet these goals, PM focuses on the patient’s stratification [82], which clusters the clinical cases into categories based on the presence or absence of specific features and potential diagnostic biomarkers (Figure 1). This stratification can be developed by large-scale multi-omics studies that integrate single-omics data obtained from patients, representing a crucial step in this new scientific approach. Single-omics datasets are integrated to compare these multiple molecular features in patients’ groups according to genomic, phenotypic, and environmental traits. To date, results obtained by single-omics are collected in international collaborative databases such as The Cancer Genome Atlas (TCGA) or Clinical Proteomic Tumor Analysis Consortium (CPTAC) [83,84]. By querying these individual datasets with various bioinformatic tools such as iCluster, iOmicsPASS, Neighborhood-based Multi-Omics clustering (NEMO), and SNF (Similarity Network Fusion), it is possible to obtain multi-omics information at various levels. Through this multi-dimensional approach, the possible outcomes are the detection of clinical subtypes, basic knowledge of cancer pathophysiology, prediction of possible therapeutic targets, and personalized follow-up.

In cancer, recent studies have been conducted emphasizing this avant-garde multidisciplinary approach. For example, miRNA and lncRNA have been demonstrated as potential biomarkers for a predictive prognosis in both HCC and prostate cancer patients according to the clustering process [85,86]. In another multi-omics study, the integrated analysis of gene copy number alterations, mutations, and proteome expression profiles enabled the identification of two distinct subtypes of pancreatic cancer based on the response to the mTOR (mechanistic target of rapamycin kinase)-targeting drugs [87].

## 3. Sequencing-Based Omics in iCCA

The use of tools such as Gene Expression Omnibus (GEO) represents a very powerful treasure to build the basis of new research projects on iCCA laying on preliminary experimental data. Over 500 datasets can be retrieved using the keywords “cholangiocarcinoma” and “genomic”, and over 200 datasets using “cholangiocarcinoma” and “transcriptome” in this repository in July 2024. Another fundamental tool is represented by TCGA, providing a detailed molecular landscape for various cancer models, including genomic (mutations, copy number variations, DNA methylation), transcriptomic (miRNAs and long transcripts, both coding and non-coding), and proteomic data. Within this project, a cohort of cholangiocarcinoma (CCA) samples has been characterized (dataset TCGA-CHOL) [88].

This dataset includes a total of 45 tissue samples, comprising 38 samples of CCA, subdivided into intrahepatic (*n* = 32), extrahepatic/hilar (*n* = 4), and mixed iCCA/HCC (*n* = 2), and 9 samples of unaffected solid tissues; clinicopathological parameters of each patient are also available for correlation analysis. At the transcriptomic level, the TCGA-CHOL dataset provides expression data of 1779 miRNAs and 20,530 long transcripts generated by RNA sequencing (RNA-seq) (Illumina) (URL https://xenabrowser.net/ accessed on 24 September 2019); 485,578 identifiers for DNA methylation analysis resulting from Illumina Human Methylation 450 are also available. All iCCA sequencing-based omics features are summarized in Table 1.

### 3.1. Genomics

In recent years, significant efforts have been made to identify genetic alterations associated with iCCA and their potential implications for prognosis and diagnosis. The advent of NGS technology has enhanced our understanding of the molecular mechanisms underlying iCCA. However, the prevalence of mutations varies across studies, likely due to tumor heterogeneity and differences in sequencing methodologies used.

Extensive genome sequencing has revealed genetic alterations of targetable oncogenes in almost 50% of iCCA patients [42] with recurrent alterations in *IDH1* [isocitrate dehydrogenase (NADP (+)) 1] and *FGFR2* (fibroblast growth factor receptor 2).

Nevertheless, other mutations frequently occurring in iCCA are on *ERBB2* (erb-b2 receptor tyrosine kinase 2), *BRAF* (B-Raf proto-oncogene, serine/threonine kinase), *EGFR* (epidermal growth factor receptor), *KRAS* (KRAS proto-oncogene, GTPase), *TP53* (tumor protein p53), *BAP1* (BRCA1-associated protein 1), *SMAD4* (SMAD family member 4), and *ARID1A* (AT-rich interaction domain 1A) [42,43,130]. Mutations in *IDH1* and *IDH2* [isocitrate dehydrogenase (NADP (+)) 2] have been reported in 10–20% of iCCA cases, with *IDH1* occurring in 7–20% and *IDH2* in 3% of these cases [131]. Gain-of-function mutations of *IDH1* are mainly located at the arginine 132 residue, specifically R132C (44%) and R132G (14%), and lead to the increase the oncometabolite d-2-hydroxyglutarate (2-HG), which is associated with epigenetic changes and defective cellular differentiation [132,133,134,135]. Additionally, *IDH* mutations affect hypoxia signaling and collagen processing and promote epithelial–mesenchymal transition (EMT) through the increased expression of ZEB1 (Zinc Finger E-Box Binding Homeobox 1) [136]. While some studies have linked *IDH* mutations to poorly differentiated CCA and clear-cell histology, others have found no association with histological grade [137]. Notably, *IDH* and *FGFR* alterations are generally mutually exclusive [138]. Aberrations in *FGFR* genes, such as amplification, single-nucleotide variants, or gene fusions, are common in various solid tumors, including iCCA. Notably, gene fusions come from translocation, chromosomal rearrangements, or interstitial deletions causing a massive and abnormal production of an active protein that acts as an oncogenic driver of malignant transformation and progression in many cancers [139]. Concerning this, it has been reported that *FGFR2* is the most frequent somatic gene fusion in iCCA and a variety of its fusion partners has been detected, suggesting that this genetic event may be a suitable candidate both for therapeutic strategies and clinical management [90,140]. *FGFR2* fusions have been observed in 10–20% of iCCA patients; they are mutually exclusive with *KRAS*/*BRAF* mutations and often co-occur with *BAP1* alterations [43,90,141]. Some reports indicate a correlation between *FGFR* fusions and improved prognosis in iCCA patients, with median cancer-specific survival significantly longer for those with *FGFR2* rearrangements (123 months) compared to those without (37 months) [142]. In the frame of the *FGFR2* alterations, Futibatinib, a covalently binding FGFR2 inhibitor, paved the way to PM in iCCA. In a recent phase 2 study, 103 patients stratified for their unresectable or metastatic *FGFR2* fusion-positive or *FGFR2* rearrangement-positive iCCA were enrolled and treated with Futibatinib [143]. After daily oral administration for 12 months, among the 43 patients with a response, 31 (72%) had responses lasting at least 6 months and 6 (14%) had responses lasting at least 12 months, without affecting quality of life. Thus, the use of Futibatinib led to measurable clinical benefit, although its effect on OS is currently under investigation in a phase 3 study.

Genomics profiling has revealed that genetic alterations in *EGFR* and *ERBBs* are implicated in different kinds of solid tumors. Both are involved in the regulation of several pathways to promote cancer progression as cell proliferation, metastasis, and angiogenesis by binding a large variety of ligands including epidermal growth factor (EGF) and transforming growth factor-α (TGFα) [144]. *EGFR* and *ERBBs’* contribution to iCCA progression is still relatively unknown. Indeed, DNA amplification of *ERBB2* and its increased expression have been observed in 0 to 73% of iCCA cases and are associated with early carcinogenic events; despite this, no point mutations in *ERBB2* have been detected to date [145,146]. On the other hand, amplification of *EGFR* and its activating point mutations are rare events in iCCA and occur in approximately 5% of patients [91]. Prognostically, *EGFR* mutations are associated with poor survival and cancer progression, while *EGFR* high expression has been identified as a negative predictor of overall survival (OS) in CCA [147].

In cancer, *KRAS* and *TP53* mutations are considered the most common oncogenic gene driver [148]. *KRAS* is involved in promoting cancer progression by reducing tumor immunogenicity [149,150]. Similarly, mutation of *TP53* causes the decrement of the onco-suppressor p53 affecting the T cell activation and consequently tumor immune escape [151]. In iCCA, *KRAS* and *TP53* have a considerable prognostic value, because mutations on these genes are associated with poor prognosis and dismal survival rate in patients [152]. Moreover, *KRAS* mutations have been linked to both iCCA and extrahepatic cholangiocarcinoma (eCCA). These include common mutations such as G/A transitions in codon 12 and less frequent ones like GGT/GAT and CCA/CAC transitions in codons 12 and 61, respectively. *KRAS*-activating mutations have been detected in 5–57% of patients with iCCA, with a higher incidence often correlating with advanced tumor stages and poor prognosis [153]. *KRAS* mutations are also considered independent predictors of worse survival rates following hepatectomy [154]. Instead, *TP53* mutations are well documented in iCCA, and numerous studies have investigated their specific incidence and types [155,156]. These loss-of-function mutations are observed in about 15% of iCCA and predominantly occur in exons 5, 6, 7, and 8, often as transitions (G to A) or, less frequently, as transversions (G to T) and are associated with poorer outcomes [152,157].

Mutations on the gene for the kinase *BRAF* are rare in biliary tract cancers, occurring almost exclusively in iCCA with an overall prevalence of 1% to 7% [158]. Specifically, activating mutations in exon 11 of *BRAF* (V600E) result in the constitutive dimerization of the BRAF protein, which activates the MAPK (mitogen-activated protein kinase) signaling pathway, which, in turn, promotes cell proliferation and inhibits apoptosis. *BRAF* V600E mutation is associated with higher TNM (Tumor Node Metastasis) stages, resistance to systemic chemotherapy, a more aggressive clinical course, and poorer survival outcomes [159,160].

*BAP1* mutations are found exclusively in patients with iCCA among all the CCA subtypes [92]. These alterations are among the most frequent in iCCA, with about 20–25% of cases exhibiting various loss-of-function mutations in this gene [42,92,137]. It has been reported that germline *BAP1* mutations may predispose individuals to iCCA. Various studies found different and conflicting prognostic meanings of *BAP1* mutations. The loss of *BAP1* showed a strong trend toward improved median survival and was associated with higher histological grade and absent lymphatic invasion [161]. On the other hand, no differences in OS or relapse-free survival (RFS) were observed between *BAP1*-mutated and *BAP1* wild-type patients who underwent radical surgery or were treated with cytotoxic chemotherapy [42,162].

Loss of SMAD4 activity is a common characteristic of gastrointestinal tumors and is most frequently observed in CCA originating in the distal common bile duct, near the pancreas—where *SMAD4* mutations are particularly prevalent [163]. In iCCA cases from Eastern countries, where liver fluke infection, viral hepatitis, and hepatolithiasis are common, *SMAD4* mutations are the most recurrent, occurring in 5 to 20% of cases [93,164]. These mutations often co-occur with *TP53* and *KRAS* mutations [93]. Additionally, loss of SMAD4 expression is more common in metastatic iCCA compared to non-metastatic cases [94,165].

ARID1A is believed to act as a tumor suppressor due to its loss-of-function mutations observed in many cancers, including gastrointestinal types, leading to a loss of protein expression [166,167]. Recent evidence links *ARID1A* mutations to CCA [168,169]. More specifically, these mutations are found in 7% to 36% of iCCA cases and 5% to 12% of eCCA cases [170]. Various molecular mechanisms, such as cell-cycle disruption, chromatin remodeling, oxidative stress damage, and DNA hypermethylation, are implicated in the relationship between *ARID1A* mutations and CCA pathogenesis [171]. However, the exact impact of ARID1A on iCCA prognosis and clinicopathologic features remains debated. Some studies suggest ARID1A has dual roles in both promoting and suppressing tumors in CCA. For instance, Yang et al. found that low ARID1A expression was linked to poorer prognosis in iCCA compared to high expression [172]. Conversely, Bi et al. reported that high ARID1A expression might correlate with a worse prognosis in iCCA [173].

Low-frequency loss-of-function mutations in iCCA are also found in *APC* (APC regulator of the WNT signaling pathway), *BRCA1* (BRCA1 DNA repair associated), *BRCA2* (BRCA2 DNA repair associated) [174,175], as well as *NTRK* fusions, which are reported in less than 1% of CCA patients [176].

To date, a limited number of studies have analyzed the patterns of chromosomal alterations in iCCA. (i.e., copy number gains and losses) by using genome-wide technologies. Just a few comparative genomic hybridizations (CGH) studies on iCCA were performed, but in many of these studies, the authors reported analyses where intrahepatic and extrahepatic tumors were merged, even including gallbladders, making a correct interpretation difficult for iCCA. The meta-analysis based on five CGH studies performed by Andersen et al. showed in iCCA frequent copy number loss on chromosome arms 1p, 4q, 8p, 9p, 17p, and 18q, while copy number gain is seen on chromosomes 1q, 5p, 7p, 8q, 17q, and 20q in at least three studies with >20% overall variation [177]. By using a high-density single-nucleotide polymorphism array, Sia et al. identified high-level amplifications in five regions, including 1p13 (9% of patients) and 11q13.2 (4%), and several focal deletions, such as 9p21.3 (18%) and 14q22.1 (36%). Such alterations combined with expression dysregulations provided the identification of two classes of iCCA associated with different outcomes [95].

Dalmasso et al. conducted a genome-wide copy-number alterations (CNAs) study using frozen material in a surgical series of 53 iCCA patients and evaluated the relationships between clinicopathological features and chromosomal abnormalities [96]. They found that the frequency rates of deleted cytobands were higher than those of amplified cytobands. More specifically, the exclusively deleted cytobands were detected on 1p36.33–1p35.1, 3p26.3–3p14.25, and 14q24.1–14q32.33. On the other hand, the exclusively amplified cytobands were reported on 1p11.2–1p41.1, 1q21.1–1q44, 2q23.1–2q35, 7p22.3–7p11.1, 7q11.1–7q36.3, and 8q23.2–8q24.3. Interestingly, the 6q deleted region was not considered to be exclusively deleted because this genomic region also showed a moderate level of amplification. Four candidate tumor suppressor genes *RUNX3* (RUNX family transcription factor 3), *ARID1A*, *ERRFI1* (ERBB receptor feedback inhibitor 1), and *mTOR*, are all located in the 1pter region and are involved in transcription and cell-cycle control. Notably, *ARID1A* and *mTOR* are functionally related to the PI3K/AKT pathway, usually overactivated in iCCA tumors.

*BAP1* and *PBRM1* (polybromo 1) are located on 3p26.3–3p14.25. *BAP1* is a tumor suppressor gene that promotes the repair of DNA double-strand breaks, and it is often mutated in several cancer types, including non-fluke iCCA; PBRM1 is involved in transcriptional activation and repression by chromatin remodeling and has been found mutated in iCCA. The deleted region 14q24.1–14q32.33 harbors part of the coding sequence of SAV1 (salvador family WW domain-containing protein 1), which inhibits the Akt-mTOR signaling in a YAP1 (Yes1 associated transcriptional regulator)-dependent manner. The gain of chromosome 1q is among the most commonly observed alterations in HCC and has also been identified in CCA. A potential oncogenic gene embedded in this region is *CHD1L* (chromodomain helicase DNA binding protein 1-like), whose dysregulation was already associated with CCA [178]. The amplified region 2q23.1–2q35 harbors the *IDH1* gene whose gain of function mutations are related to alterations of DNA methylation in iCCA [91,137,179]. In iCCA, the entire chromosome 7 or parts of it (i.e., 7p22.3–7p11.1, q11.1–7q36.3) may be amplified [120,180]. Interestingly, many proto-oncogenes, such as *EGFR*, *MET* (MET proto-oncogene, receptor tyrosine kinase), and *BRAF*, are located in chromosome 7 and their gain of function mutations or overexpression was already reported for iCCA [181,182,183]. Finally, the amplified region 8q24.21 harbors the *MYC* (MYC proto-oncogene, bHLH transcription factor) gene, the oncogenic transcription factor promoting the expression of several proto-oncogenes. Its overactivation is often reported in multiple carcinomas, including iCCA [184,185].

### 3.2. Epigenomics

In the omics era, cutting-edge approaches have revealed alterations not only in gene sequences but also in the epigenetics landscape in iCCA. To date, it has been observed that iCCA epigenomics dysregulation arises from mutations in epigenetic regulators, changes in chromatin remodeling, and DNA methylation rates.

Although the causes of these abnormalities and their effects on iCCA pathobiology are still unclarified, the epigenomics profile may be added to the more detailed knowledge about the wide iCCA heterogeneity to develop sensitive biomarkers to improve the clinical approaches to this dismal malignancy.

DNA methylation is an epigenetic mechanism correlated with gene expression, and aberrant alterations in DNA methylome are detected in CCA [186]. In particular, abnormalities in the DNA methylation rate of CpG islands (CGIs) of tumor suppressor gene (TSG) promoters causing the induction of oncogenes pathways that may contribute to CCA development have been observed [187,188]. Regarding this, Peng et al. [98] reanalyzed the DNA methylation GEO-dataset GSE89803 [189], integrating the data of about 83 iCCA samples and 4 unaffected tissues in order to identify key hub genes relevant to the progression of iCCA. A co-expression network was built according to 43 tumor samples with stages I and IV, leading to the identification of 5502 genes showing the largest variance across the analyzed tissues; hierarchical clustering based on these genes resulted in the removal of one outlier sample. The hub genes of the network included *SLC2A1* (solute carrier family 2 member 1), *CDH3* (cadherin 3), and *EFHD2* (EF-hand domain family member D2), whose expression increased with tumor stage and was associated with a lower survival rate. Similarly, the expression of *FAM171A1* (family with sequence similarity 171 member A1), *ONECUT1* (one cut homeobox 1), and *PHYHIPL* (phytanoyl-CoA 2-hydroxylase-interacting protein-like) decreased with tumor stage and was associated with survival. The authors suggested that the dysregulation of the hub genes may be associated with an altered pattern of DNA methylation at the promoter regions. Analyzing the dataset GSE89803 [189], they found that the loci cg15026696 and cg06972969 were associated with the reduced expression of *EFHD2* and *PHYHIPL*, respectively. Also, survival analysis revealed that cg15026696 hypomethylation and cg06972969 hypermethylation were correlated with lower survival rates [98].

Dragomir and collaborators [100] analyzed several datasets including iCCA, pancreatic ductal adenocarcinoma (PDAC), normal bile duct, and normal pancreas samples to identify classifiers able to discriminate the two similar tumors. Specifically, two TCGA [88,190] and five GEO datasets (GSE155353 [191], GSE49149 [192], GSE89803 [189], GSE201241 [97], GSE156299 [193]) were used as a discovery cohort. Methylation data relative to the 2048 most variable CpG sites allowed samples to be clustered according to their tissue of origin, with PDAC samples and normal bile duct being part of the same cluster and iCCA samples being divided into two clusters. By using three different machine learning models (random forest, support vector machine, and neural network), the same data were analyzed to identify a classifier. Among the 2048 CpG sites, regulating the expression of 1074 unique genes, 359 showed altered methylation levels comparing iCCA and PDAC, with all sites being hypermethylated in iCCA, while only cg15073906 (mapping within the 3′UTR of *RALGPS2*) and cg03019505 (mapping within the body of *TFIP11*) were hypermethylated in PDAC. Looking at the RNA of the 155 genes modulated by the dysregulated methylation sites, 107 were significantly differentially expressed (DE) between iCCA and PDAC, with 79 (73.83%) transcripts showing a dysregulation trend coherent with the methylation level. A validation cohort was established, grouping 361 samples deriving from the previous datasets plus others (GSE149250 [194], GSE32079 [132], GSE49656 [137]). In general, the neural networks showed the best performance. Another validation was performed on an independent in-house cohort. Primary and metastatic PDAC showed little differences, while lots of sites showed altered methylation patterns between iCCA and PDAC. The neural network classifier showed a good performance and outperformed an immunohistochemistry-based signature comprising ANXA1 (annexin A1) and ANXA10 (annexin A10) in recognizing iCCA precursor lesions and pCCA, both showing high similarity with the tumor [100].

Through whole-genome bisulfite sequencing (WGBS) performed on iCCA patients’ tissues, Chen and colleagues [101] proposed the concept of prognostically methylated regions (PMRs), consisting of prognostically methylated CpG (PMCs) sites showing a concordance index (C-index) for an OS higher than 0.6. In the discovery cohort, 652,123 CpG sites were defined as PMCs, with 35,023 PMRs identified within them. The efficiency of these sites as biomarkers was tested in the validation cohort, showing an overlapping for 47% PMCs and 78.8% PMRs. Given the higher percentage of validated sites, the authors suggested that PMRs perform better as prognostic biomarkers. A prognostic methylation score (PMS) was then calculated by using the random forest machine learning method and the least absolute shrinkage and selector operation (LASSO) Cox algorithm was applied to build a prognostic model using the candidate PMRs. Of the 362 PMRs selected, 14 regions were further picked out by the LASSO Cox algorithm. These regions are mapped within the coding sequences of both protein-coding and non-coding genes. Application of the PMS in the discovery cohort showed a successful classification of 54.9% into the PMS-low group and 45.1% into the PMS-high group, with a better OS for the PMS-low group. Similar results were obtained in the validation cohort, with 58.8% and 41.1% successful classification in the PMS-low and PMS-high groups, respectively; again, a better OS for the PMS-low group was observed. In both cohorts, the increase in PMS score was accompanied by a high number of patients dying shortly after the surgery. The PMS score also showed a good performance in identifying appropriate candidates for adjuvant therapy [101].

Together with DNA methylation, another key epigenetic component in cancer development is given by histone modifications. Changes to histone tails cause abnormalities in chromatin remodeling with the result of gene expression alterations. Several histone modifications are involved in cancer genesis [195]. Nucleosome rearrangement affects cancer metastasis [196], and it is considered a useful marker for early estimation of the response to therapy in some kinds of tumors [197].

He and colleagues [99] performed epigenetic profiling based on the analysis of nucleosome accessibility with histone modification states in iCCA and normal biliary cell lines by chromatin immunoprecipitation-sequencing (ChIP-seq). Tumor cells showed reduced levels of H3K4me1, H3K4me3, and H3K27ac compared to normal cells; reduced enrichment of these modifications was observed in several genes, including the *CCND2* (cyclin D2) locus. Using H3K27ac levels, indicators of active enhancers, super-enhancers (SEs) specific to iCCA cells were discovered, explaining the peculiar gene expression profile of tumor cells. Sequence analysis of the observed chromatin modifications suggested that transcription factors of the AP-1 family may be involved in the regulation of gene expression in iCCA cells. Nucleosome occupancy was also evaluated by micrococcal nuclease-sequencing (MNase-seq), resulting in a more stable nucleosome profile in iCCA cells for downregulated genes, while enrichment in variable nucleosomes was observed for upregulated genes. The authors suggested a role for JUN (Jun proto-oncogene, AP-1 transcription factor subunit) in regulating the expression of iCCA-related genes and apoptosis of tumor cells. Accordingly, immunostaining in 41 tumor and unaffected adjacent tissues showed reduced levels of JUN; downregulation at the transcript level was also observed for *JUN*, *FOS* (Fos proto-oncogene, AP-1 transcription factor subunit), and *CCND2*. Finally, JUN and FOS expression levels affected OS and progression-free survival in the same cohort [99].

Recently, Liao et al. [102] performed WGBS on 331 iCCA samples and 9 unaffected tissues. This analysis led to the identification of four subtypes of iCCA (S1–S4) in which a general hypomethylation was observed in comparison to normal tissues, especially in the S2 subgroup. Specific clinicopathological features were associated with each subgroup, with S2 and S3 patients showing a poorer OS than the S1 and S4 ones. Interestingly, S4 patients showed the best OS and a methylation level comparable to unaffected controls. Further analyses allowed a deeper characterization of specific genomic sites subjected to altered methylation in tumor and normal tissues, with variable methylation patterns of partially methylated domains (PMDs) and highly methylated domains (HMDs) within each iCCA subgroup. Looking at CGIs, hypermethylation was observed through all subgroups: S1 and S4 samples were characterized by higher CGI methylation than S2 and S3, while unaffected tissues showed the lowest CGI methylation level, coherently with most solid tumors [102].

### 3.3. Transcriptomics

Transcriptomics approaches allow the monitoring of transcriptome changes potentially associated with cancer. Together with genomics and proteomics, it is a widely used branch in omics studies to classify tumors, aiming to predict prognosis or response to therapies.

In the iCCA context, three classifications were proposed between 2012 and 2013: Andersen et al. proposed a classification based on transcriptomic profiles and mutations in *KRAS*, *EGFR*, and *BRAF* genes [198]; Oishi et al. grouped iCCA tumors according to mRNA and miRNA expression [199]; and Sia et al. proposed a categorization based on gene expression profiles and activation of specific signaling pathways [95]. Job and colleagues [103] performed gene expression profiling by Affymetrix array on 78 iCCA formalin-fixed, paraffin-embedded (FFPE) samples and 31 adjacent unaffected tissues. The gene expression profiles were used to assign each tumor tissue to the appropriate subgroup according to the three reported classifications, and the correlation among these was evaluated: Andersen’s and Oishi’s classifications, both based on frozen samples, were strongly concordant, while the correlation with Sia’s grouping, based on the analysis of FFPE tissue samples, was moderate [103].

Ahn and collaborators [104] identified two molecular iCCA subtypes with distinct molecular and clinical features. Performing RNA-seq, they found 2379 DE genes that allowed tumor samples to cluster in two subclasses associated with OS. Subclass A is associated with a better prognosis, in which viral hepatitis and cholangiolar-type pathology are more frequent. On the contrary, subclass B shows a poor prognosis presenting a high frequency of *KRAS* mutations and being rich in inflammation-related pathways [104].

Silvestri et al. [105] collected transcriptomic data generated by RNA-seq and microarray publicly available at TCGA (TCGA-CHOL) [88] and GEO (GSE132305 [200], GSE32225 [95], GSE26566 [198], GSE89749 [189], GSE32879 [199], and GSE57555 [201]). These datasets included both iCCA and eCCA samples and were analyzed aiming to validate a classification of CCA based on gene expression and identify prognostic biomarkers. Comprehensively, a total of 340 iCCA and 203 eCCA samples were analyzed. Unsupervised clustering analysis revealed the existence of four subgroups of tumors (A-D), showing significant differences in the distribution of dysregulated genes (both up- and downregulated). Patients grouped in subgroup A are characterized by tumors devoid of immune cell infiltration and with high expression levels of KRAS, EMT, and apoptosis markers. These patients, indeed, have the worst prognosis and relapse-free survival. On the other hand, patients in subgroups B and C present a strong immunological component directing them to a better prognosis; nevertheless, they have aberrant activation on several cancer-associated pathways such as RTK-RAS-PIK3K and TGF-β. Sets of 19 and 21 genes for iCCA and eCCA, respectively, were chosen as signatures to be validated in an independent cohort represented by the dataset EGAD00001001693, retrieved from The European Genome-phenome Archive (EGA) and consisting of 131 samples (102 iCCA vs. 29 eCCA) profiled by RNA-seq, confirming the results obtained in the training set. The association of specific subgroups with OS was reported, suggesting that the biological characteristics of the primary tumors affect the prognosis of the patients [105].

Transcriptomic approaches may be focused on coding transcripts and/or non-coding transcripts. While the expression of mRNAs and miRNAs has been widely investigated in the last decades, lncRNAs and circular RNAs (circRNAs) have been discovered more recently, and fewer data are available in the literature to date, especially for some cancer models. Often, transcriptomic studies aim to identify the DE transcripts and infer from them the molecular basis of carcinogenesis.

In 2017, Chen and collaborators [106] reanalyzed two datasets from GEO reporting transcriptomic data about mRNAs (GSE32879) and miRNAs (GSE32957) [199]. In both datasets, 16 iCCA and 7 paired non-cancerous liver tissue samples were considered; Affymetrix arrays investigated mRNA expression, while the NanoString microRNA platform was used for miRNAs. A total of 2327 DE mRNAs (1113 up- and 1214 downregulated) and 70 DE miRNAs (65 up- and 5 downregulated) were identified; also, 623 genes undergoing alternative splicing were detected. For DE miRNAs, predicted targets were retrieved, finding 63 overlaps with DE mRNAs and transcripts showing alternative splicing; also, 243 miRNA-mRNA pairs were identified, with 52 showing an opposite trend of dysregulation for miRNA and mRNA [106].

Yang and colleagues [107] analyzed expression data from seven pairs of iCCA and unaffected tissues retrieved from GEO (GSE63420) [89]. A total of 230 DE lncRNAs (97 up- and 133 downregulated) were identified, with *RP11-328K4.1*, *LINC01093* (long intergenic non-protein coding RNA 1093), *LINC00844* (long intergenic non-protein coding RNA 844), *RP11-372E1.4*, and *ITIH4-AS1* (ITIH4 antisense RNA 1) being the most significantly downregulated, and *RP11-532F12.5*, *AC016735.1*, *RP11-284F21.7*, *LINC01123* (long intergenic non-protein coding RNA 1123) and *AC013275.2* the being most significantly upregulated lncRNAs. Among coding transcripts, 2220 mRNAs showed differential expression (640 down- and 1580 upregulated). Both DE lncRNAs and mRNAs clustered together tumor or unaffected samples [107].

Peraldo-Neia et al. [108] performed gene expression profiling by the Agilent microarray platform in 11 pairs of primary (PR) and recurrent (REC) iCCA tumors collected from the same patient. Unsupervised hierarchical clustering showed that, in some cases, paired PR and REC tumors gave markedly different gene expression profiles, coherent with the high heterogeneity of the tumors. In total, 315 genes were significantly dysregulated (65 down- and 250 upregulated) in REC compared with PR tumors. A signature including the 24 top dysregulated genes (10 down- and 14 upregulated) able to discriminate REC from PR tumors was identified. This signature was also validated in an independent cohort of 13 samples (3 REC vs. 10 PR iCCA tumors), showing that nine genes (*NDST2*, *DAO*, *FANCG*, *CARD9*, *FOXJ2*, *SEMA3B*, *GDAP1L1*, *TRIOBP*, *PFKM*) discriminate REC from PR tumors [108]. Among them, *FANCG* (FA complementation group G) is associated with survival in the TCGA-CHOL [88] dataset.

Cao and collaborators [109] used a transcriptomic approach focused on miRNA expression data available at TCGA-CHOL [88]. By analyzing expression data from 36 CCA samples and 9 unaffected tissues, 100 DE transcripts (54 up- and 46 downregulated) were identified. Among them, three miRNAs, specifically, mir-10b, mir-22, and mir-551b were significantly associated with OS. Aiming to identify a biomarker signature, a score based on low or high expression of each OS-related pre-miRNA was calculated for each patient. This score was used to divide the 36 patients into high- and low-risk groups, both significantly associated with OS. According to the multivariate analysis, the pre-miRNA signature was an independent prognostic factor for CCA. The authors validated these results in another miRNA expression dataset available at GEO (GSE53870), confirming the accuracy of the signature; however, the discrepancy between the data analyzed by Cao and collaborators (pre-miRNA expression) and those included in this dataset (mature miRNA expression) must be noted. Finally, the authors focused their attention on miR-551b for in vitro assays: ectopic expression of miR-551b (-3p, according to the sequence reported in the Methods Section) in the iCCA epithelial cell line (HuCCT-1) reduced cell proliferation and colony formation, and also increased apoptosis [109].

Ye and colleagues [110] analyzed the dataset GSE107943 (30 iCCA vs. 27 unaffected tissues) [202] and identified DE transcripts. Since the adjacent unaffected tissues consisted of liver samples, liver-specific genes were identified using the dataset GSE26566 (including 6 normal intrahepatic bile duct samples and 59 liver tissues) [198] and were removed from the results, obtaining 1643 DE transcripts (1098 up- and 545 downregulated). The DE transcripts were used to build a weighted gene co-expression network (WGCN), in which seven co-expressed modules were identified. Five modules were associated with clinicopathological parameters of iCCA patients, such as tumor class, hepatitis B virus (HBV) infection, tumor recurrence, tumor-associated mortality, serum carbohydrate antigen 19-9 (CA19-9) expression, and disease-free survival (DFS). A total of 63 genes from two modules were selected as core genes because of their association with four clinicopathological parameters. A protein–protein interaction (PPI) network was built on the 63 core genes, with *CCNB1*, *CDC20*, *CDCA8*, *CDK1*, *CEP55*, *KIF2C*, *TOP2A*, and *TPX2* representing the hub genes [110]. The diagnostic performance of the hub genes singularly was evaluated in the dataset GSE119336 (15 iCCA and 15 paired unaffected tissues) [203], showing excellent accuracy, with each area under the curve (AUC) value greater than 0.9.

Kang et al. [204] applied a similar approach using available expression data from Sequence Read Archive (SRA) and TCGA. The DE transcripts were used to build a lncRNA–miRNA–mRNA competing endogenous RNA (ceRNA) network, where 60 co-expressed DE mRNAs associated with iCCA were detected. Among them, six hub genes were identified in *FOS* (Fos proto-oncogene, AP-1 transcription factor subunit), *IGF1R* (insulin-like growth factor 1 receptor), *HGF* (hepatocyte growth factor), *IGF2* (insulin-like growth factor 2), *FOXO1* (forkhead box O1), and *NTF3* (neurotrophin 3). The expression of these six mRNAs was evaluated in the TCGA-CHOL dataset, showing upregulation for *IGF1R* and downregulation for *FOS*, *HGF*, *IGF2*, *FOXO1*, and *NTF3*. Among all DE transcripts, association with OS was only observed for lncRNA *MME-AS1* (MME antisense RNA 1) and miR-182 [204].

Xia and collaborators [111] performed a pathway enrichment analysis on DE lncRNAs and mRNAs in iCCA and unaffected tissues revealing their involvement in the Hippo pathway. Consistently, YAP1 and its homologous transcriptional coactivator, TAZ, (WWTR1, WW domain containing transcription regulator 1) were upregulated in iCCA tissues. LncRNAs co-expressed with YAP1 and DE miRNAs targeting its mRNA were identified to build a YAP1-centered ceRNA network, which included five lncRNAs and five corresponding miRNAs (RP11-57A19.2 and miR-27a-3p, CTD-2132N18.2 and miR-199a-3p, RP11-774O3.3 and miR-200a-3p, RP11-375I20.6 and miR-141-3p, CRYM-AS1 and miR-16a-5p). The 5 lncRNAs were confirmed as upregulated in 20 pairs of iCCA and unaffected tissues. Because it showed the highest expression levels, *RP11-375I20.6* was further investigated and named prognosis-associated intrahepatic cholangiocarcinoma lncRNA (lncRNA-*PAICC*). This lncRNA was mainly localized within the cytoplasm and predicted to have no coding potential. Overexpression of lncRNA-*PAICC* and *YAP1* was confirmed in another independent cohort consisting of 76 pairs of iCCA and unaffected tissues; the two transcripts also showed a significant positive correlation of expression. In the same tumor tissues, miRNA expression was evaluated, showing a negative correlation of expression between the lncRNA and miR-141-3p and miR-27a-3p. Clinicopathological parameters such as tumor number, tumor size, vascular invasion, and OS were associated with lncRNA-*PAICC* levels. Ectopic expression or knockdown of the lncRNA altered cell proliferation, colony formation ability, migration, and invasion of iCCA cell lines. Also, the silencing of lncRNA-*PAICC* induced the reduction in *YAP1* mRNA and protein levels, while modulating the expression of the lncRNA-induced changes in miR-141-3p and miR-27a-3p levels. The effective binding of the two miRNAs to the lncRNA sequence was validated by luciferase assay and RNA immunoprecipitation (RIP), suggesting the role of miRNA sponge for lncRNA-*PAICC*. In vivo, lncRNA-*PAICC* was shown to participate in tumor growth [111].

Li and coworkers [112] performed a comprehensive transcriptomic landscape integrating four GEO datasets (GSE132305 [200], GSE89749 [189], GSE76297 [205], and GSE26566 [198]) on iCCA and eCCA, and their unaffected counterpart in order to have a better classification based on molecular and etiological features. Among the upregulated transcripts, *ARHGAP21* (Rho GTPase activating protein 21) was the only one associated with both OS and DFS; similarly, *SCP2* (sterol carrier protein 2), *UBIAD1* (UbiA prenyltransferase domain containing 1), *TJP2* (tight junction protein 2), *RAP1A* (RAP1A, member of RAS oncogene family), *HDAC9* (histone deacetylase 9), *FKBP2* (FKBP prolyl isomerase 2), *MRPL2* (mitochondrial ribosomal protein L2), and *MRPL27* (mitochondrial ribosomal protein L27) were downregulated and associated with OS and DFS [112].

Tumor heterogeneity may be investigated by omics approaches, also including transcriptomic studies. In 2018, Rhee and collaborators [113] focused on a new subtype of iCCA named cholangiolocellular carcinoma (CLC), characterized by a resemblance with cholangioles involving more than 90% of the tumor, often the presence of focal areas of HCC at the peripheric zone of the tumor mass and the association with viral hepatitis. To identify a signature for cholangiolocellular differentiation (CD), transcriptomic profiles of 27 iCCA cases (10 with vs. 17 without CD) were performed by Illumina microarrays. Expression data led to the clustering of all iCCA with CD together, except for one sample. The CD signature included 794 DE transcripts (486 up- and 308 downregulated). Among genes related to iCCA with CD, *CRP* (C-reactive protein) was the most upregulated, while *S100P* (S100 calcium-binding protein P), *TFF1* (trefoil factor 1), *AGR2* (anterior gradient 2, protein disulfide isomerase family member), *CLDN18* (claudin 18), *KRT17* (keratin 17), and *CTSE* (cathepsin E) were characteristic of iCCA without CD. The two subgroups of iCCA showed different clinical outcomes, with iCCA cases with CD being characterized by better prognosis and time to recurrence. These observations were confirmed on the dataset GSE26566, used by Andersen and collaborators [198] to propose an iCCA signature; in this dataset, the CD signature confirmed its association with a better prognosis, despite only 66 genes (8% of the CD signature) overlapping. Similarly, the CD signature was validated on the GSE32225 dataset [95], used to generate the Sia signature. A comparison of the three studies showed that only seven transcripts were present in all the signatures, namely, *SCTR* (secretin receptor), *TMEM156* (transmembrane protein 156), *WNK2* (WNK lysine deficient protein kinase 2), *SERPINB5* (serpin family B member 5), *KRT17*, *ANLN* (anillin, actin-binding protein), and *S100P*. Since some genes expressed in iCCA without CD were previously associated with PDAC (such as *CLDN18*, *KRT17*, and *CTSE*), the similarity between iCCA and PDAC at the gene expression level was also investigated using the dataset GSE36924 [206], including 90 PDAC samples. A close similarity in gene expression was observed between PDAC and iCCA without CD. Interestingly, the CD signature was downregulated in 80 out of 90 PDAC tumors [113].

Zhang et al. [114] characterized eight samples (four treatment-naïve and one recurrent tumor sample, and three adjacent unaffected tissues) by single-cell RNA-seq (scRNA-seq) performed on 56,871 unselected viable cells. Gene expression profiles allowed the identification of several subsets of tumor, immune, and stromal cells, among which malignant cells showed a high degree of inter-tumor heterogeneity. Specific markers were used to classify cells into specific classes, subdivided into six main subclusters through the reclustering of all the malignant and normal epithelial cells. Each subcluster was characterized by high expression levels of specific genes. In particular, *SPINK1* (serine peptidase inhibitor Kazal type 1) was associated with survival and involved in the regulation of tumor sphere propagation, colony formation, invasion, and drug resistance. Tumor heterogeneity and specific transcriptomic signatures also characterized tumor-infiltrating regulatory T cells (Tregs) and fibroblasts [114].

Xiang and coworkers [115] combined a genomic approach with a transcriptomic one. The authors enrolled iCCA patients, collecting three to five tumor regions from each one. For RNA-seq, 54 tumor regions from 12 patients were analyzed. Unsupervised hierarchical clustering showed that, based on expression data, inter-tumor heterogeneity is higher than intratumor heterogeneity. RNA intra- and inter-tumor heterogeneity scores were calculated, resulting in the generation of four RNA heterogeneity quadrants. For each one, the prognostic score was computed and showed that the expression of many pan-cancer prognostic genes was heterogeneous within the tumor regions collected from a single patient and between different patients. This observation suggests that tumor heterogeneity makes identifying prognostic markers for iCCA a challenging task. Four representative tumors were selected for scRNA-seq, which allowed the discrimination of malignant from non-malignant cells and determined the differences in immune cells between patients affected by IDH-mutated and IDH wild-type tumors. These results suggest that identifying IDH-mutated patients may be clinically useful for patient management [115].

Zhou and collaborators [116] reanalyzed expression data from GSE26566 (104 CCA and 6 paired unaffected tissues) [198] and GSE32225 (149 iCCA and 6 paired unaffected tissues) [95], identifying respectively 1613 (710 up- and 903 downregulated) and 998 (432 significantly up- and 566 downregulated) DE transcripts. The robust rank aggregation (RRA) method was used to integrate DE transcripts. A PPI network including 202 interactions was built on these DE transcripts. Mutations of TCGA-CHOL samples were also analyzed. By intersecting the lists of DE transcripts and mutated genes, the authors focused their attention on INTS8 (integrator complex subunit 8), which showed the best diagnostic performance in receiver operating characteristic (ROC) curve analysis. INTS8 protein levels were increased in CCA tissues (155 CCA vs. 5 paired peri-tumoral tissues). The expression of INST8 was associated with specific patterns of tumor-infiltrating immune cells, age, and tumor grade [116].

Wang and colleagues [93] published a similar work. The authors analyzed the mutational landscape of 1481 iCCA tumors and classified them into three genetic clusters. Independently, nine gene expression datasets (TCGA-CHOL [88], GSE26566 [198], GSE32225 [95], GSE76297 [205], GSE66255 [113], GSE89748 [189], E-MTAB-6389 [103], GSE107943 [104], GSE107100 [108]) were collected. The genetic clusters showed differences in mutations, clinicopathological features, clinical outcomes, histology, sensitivity to therapy, and, obviously, gene expression profiles. In particular, 52 genes showed differential expression between Cluster1A and Cluster2, with the two most dysregulated genes *S100P* and *KRT17* being already considered prognostic markers in Andersen’s [198], Sia’s [95], and CD [113] signatures. Based on these genes, upregulated at both mRNA and protein levels, the authors proposed a clinicopathological (CP) score calculated considering the expression of *S100P*, *KRT17*, and *CA19-9*, a carbohydrate antigen known to be cluster-specific and associated with prognosis and histology. A low CP score classified tumors into Cluster2, while a high CP score defined Cluster1A tumors. This score showed a better diagnostic performance compared with CA19-9 alone [93].

Liao and collaborators [117] performed circRNA sequencing on 30 iCCA tissue samples, among which 15 were obtained from patients with extrahepatic post-operative metastases and 15 from patients without post-operative metastases. Four circRNAs showed increased expression in metastatic tumors, namely, *circZNF215* (zinc finger protein 215), *circBNIP3L* (BCL2 interacting protein 3 like), *circCD109* (CD109 molecule), and *circPLOD2* (procollagen-lysine,2-oxoglutarate 5-dioxygenase 2). Among them, *circZNF215* showed the strongest upregulation and highest expression in iCCA cell lines HuCCT1, RBE, and HCCC9810, and was chosen for further investigations. This circRNA derives from exons 2, 3, and 4 of the transcript variant 1 of the *ZNF215* locus (transcript ID: ENST00000278319.10) and was preferentially localized in the cytoplasm of iCCA cells. In vitro and in vivo experiments including the silencing and overexpression of *circZNF215* showed its involvement in tumor growth and metastatization in iCCA. Also, *circZNF215* overexpression altered the expression of 344 genes (159 up-and 185 downregulated), inactivated PTEN (phosphatase and tensin homolog) by oxidation, and led to the triggering of the PI3K/AKT pathway. The molecular function of *circZNF215* was investigated by RIP assays, showing no binding with *AGO2* (argonaute RISC catalytic component 2), and therefore excluding the miRNA sponge function, while direct interaction with the antioxidant enzyme PRDX1 (peroxiredoxin 1) was proved. Specifically, *circZNF215* directly interacts with PRDX1, preventing its binding to PTEN, thus leading to its oxidation and consequent inactivation. Interestingly, *circZNF215* downregulation increased the efficacy of ipatasertib treatment, suggesting this circRNA may represent a potential therapeutic target for iCCA [117].

Zhang et al. [118] collected expression data on circRNAs, miRNAs, and mRNAs from three GEO datasets: GSE181523 (7 iCCA vs. 7 paired unaffected tissues) [207], GSE32957 (16 iCCA vs. 5 unaffected tissues) [199], and GSE61850 (5 iCCA vs. 5 paired unaffected tissues). A total of 173 DE circRNAs (69 up- and 104 downregulated), 58 DE miRNAs (30 up- and 28 downregulated), and 4437 DE mRNAs (2234 up- and 2203 downregulated) resulted from the analysis. By comparing the list of DE miRNAs and the list of miRNA-response elements (MREs) present within the sequences of DE circRNAs, seven miRNAs were selected (miR-34c-5p, miR-516a-3p, miR-513a-3p, miR-1261, miR-512-3p, miR-199b-5p, and miR-1183). Predicted targets of these miRNAs were retrieved and compared with the list of DE mRNAs, selecting the 1864 common transcripts. These targets were then filtered to select only 148 immune-related genes. Among them, *IL23R* (interleukin 23 receptor) was associated with prognosis and immune infiltration within the tumor. Therefore, a ceRNA network centered on *IL23R* was computed. Among the seven miRNAs previously selected, miR-512-3p and miR-1183 targeted *IL23R* and were included in the network together with the circRNAs bearing their corresponding MREs, namely, *hsa_circ_0050898*, *hsa_circ_0037100*, and *hsa_circ_0016956*. In parallel, a PPI network based on the 148 immune-related genes interacting with IL23R was also built. The association of IL23R with OS and DFS was confirmed by using the expression data from TCGA-CHOL [88]. Downregulation of *IL23R* mRNA was observed, despite no details on the analyzed samples being given [118].

Another important application for single-omics approaches relies on the identification of potential biomarkers to be used in clinical practice for diagnostic, prognostic, or therapeutic purposes. In this case, the most appropriate samples are represented by body fluids, which allows non-invasive analyses useful for the clinical management of the patients to be performed. Similar approaches were applied to several cancer models, including iCCA.

In 2016, Correa-Gallego and collaborators [119] applied a transcriptomic approach, namely, RNA-seq, to iCCA FFPE tumor (*n* = 12) and unaffected (*n* = 11) tissues to identify DE miRNAs, successively validating them in plasma samples. The analysis of FFPE samples showed 262 dysregulated miRNAs (128 up- and 134 downregulated), among which the most upregulated (miR-21, miR-34c, miR-200b, and miR-221) were validated by Real-Time PCR in an independent cohort of FFPE samples (10 iCCA vs. 5 unaffected tissues). A total of 32 plasma samples (25 iCCA of different grades vs. 7 unaffected patients) were analyzed, confirming the upregulation of miR-21 and miR-221. In particular, plasmatic miR-21 showed excellent diagnostic performance, with an AUC of 0.94, and was upregulated in poorly differentiated tumors [119].

### 3.4. Epitranscriptomics

Alterations in both structure and expression (i.e., translation) of mRNAs play a key role in affecting gene regulation in a post-transcriptional manner. These phenomena are called epitranscriptomics modifications.

To date, although epitranscriptomics is a new field still to explore, recent advances in NGS have allowed RNA chemical modification sites to be detected and mapped, highlighting how they undergo different fates dynamically affecting gene expression. The most frequent epitranscriptomics modifications include methylation, generating N6-methyladenosine (m6A), N6,2′-O-dimethyladenosine (m6Am), N1-methyladenosine (m1A), 5-methylcytosine (m5C), and 5-hydroxymethylcytidine (hm5C), or RNA editing, including adenosine to inosine (A to I) conversion [208,209]. RNA’s methylations are largely detected in liver disease, suggesting their potential involvement as prognostic and therapeutic targets [208].

Several techniques specifically implemented to study RNA modifications have been proposed, but relatively little is known about the role of epitranscriptomics modifications in iCCA to our knowledge. Some evidence has been reported, despite applying non-specific techniques.

In 2022, Zhang and collaborators [120] analyzed the genomic alterations affecting iCCA and pCCA, reporting that both mutation and CNA burdens are higher in iCCA. Important regulators of m6A methylation such as *RBM10* (RNA binding motif protein 10) and *METTL14* (methyltransferase 14, N6-adenosine-methyltransferase non-catalytic subunit) were enlisted among the mutated genes, with recurrent mutations affecting *METTL14* at the level of the same amino acid residue (p.R298H and p.R298C); similarly, CNAs were observed in chromosomic regions in which genes involved in DNA and chromatin methylation such as *DNMT3A* (DNA methyltransferase 3 alpha) and *EZH2* (enhancer of zeste 2 polycomb repressive complex 2 subunit) map. METTL14 expression was reduced both as mRNA and protein in 69 pairs of CCA and unaffected tissues, and its downregulation was associated with poor survival. Coherently, m6A methylation of total RNA was significantly reduced in CCA, particularly in tumors expressing low levels of *METTL14*. Functional assays showed the role of the *p.R298H* mutation in m6A modification ability and cancer-related processes in vitro and in vivo. Methylated RNA immunoprecipitation sequencing (MeRIP-Seq) showed that the *p.R298H* mutation affected m6A modifications on the target *MACF1* (microtubule actin crosslinking factor 1) [120].

Epitranscriptomics in iCCA was evaluated with different methodologies by Gao and collaborators [203], who recently applied a transcriptomic approach including whole-exome and transcriptome sequencing to identify potential RNA editing sites, which were later validated by Sanger sequencing. The discovery set included 15 patients (iCCA vs. paired unaffected tissues), while validation with Sanger was performed in a cohort of 26 patients. This analysis led to the identification of 25 editing sites (14 nonsynonymous and 11 synonymous sites) within coding regions, confirming A to I as the most frequent event. The authors evaluated the frequency of editing events in the discovery cohort, showing that the RNA editing phenomenon is increased in iCCA compared to unaffected tissues. The expression of *ADAR1* (ADAR, adenosine deaminase RNA specific) and *ADAR2* (ADARB1, adenosine deaminase RNA specific B1), the key regulators of A to I editing, was evaluated using RNA-seq data from the discovery cohort. *ADAR1* was upregulated in iCCA samples and showed higher expression levels than *ADAR2*. This upregulation was confirmed at the mRNA level in the validation cohort and the dataset GSE26566 [198]. At the protein level, increased expression of ADAR1 was detected by immunohistochemistry in a second validation cohort consisting of 137 iCCA compared with paired unaffected tissues. ADAR1 expression was positively correlated with the increased RNA editing events, suggesting that the dysregulation of this key enzyme sustains the altered editing pattern. CNA analyses in the discovery cohort, the GSE49666 [137] and TCGA-CHOL [88] datasets, revealed that the *ADAR1* locus (chr 1q22) was amplified in 27–55% of iCCA patients, and this genomic gain was positively correlated with its mRNA levels in iCCA and other tumors. Functional in vitro assays proved that ADAR1 was implicated in cell growth, colony formation, migration, and invasion. Also, association with OS and DFS in the second validation cohort was reported. The authors focused on a specific editing event, namely, A22G in KPC1 (RNF123, ring finger protein 123), showing that this may be regulated by ADAR1 and was involved in cancer-related processes, both in vitro and in vivo [203].

## 4. A Landscape Still Undiscovered: Proteomics and Metabolomics of iCCA

Although iCCA is mostly studied through genomics and transcriptomics profiles, these omics approaches illustrate the upstream events in the development of this malignancy, leaving the downstream outputs largely unexplored. Therefore, proteomics and metabolomics are becoming fields of increasing interest to broaden the characterization of iCCA (Table 1) [210].

### 4.1. Proteomics

In iCCA, TME plays a predominant role in contributing to the complexity of this biliary malignancy. The iCCA hallmark is a hypovascularized and high desmoplastic stroma responsible for rapid and progressive invasive growth. Furthermore, the cellular landscape displays a strong diversity in malignant, immune, and stromal cells, which, in turn, results in a crosstalk that reciprocally influences their behaviors [114]. Consequently, a comprehensive understanding of this peculiar and complex TME and the ensuing molecular events has been largely addressed in recent years.

In support of this goal, proteomics approaches may allow aberrant proteins secreted by TME and released into various kinds of body fluids, such as extracellular fluid (ECF), blood, and urine, to be identified, providing a good-non-invasive analysis for the early detection of biomarkers of iCCA and therapeutic purposes [40,211,212].

In this peculiar TME, cancer-associated fibroblasts (CAFs) play a crucial place in promoting desmoplastic reaction that leads to aberrant production of extracellular matrix (ECM) frequently correlated with poorer survival outcomes [213]. Therefore, proteomics studies have been focused on investigating the composition and role of the highly thickened ECM in iCCA scenarios.

A matrisome analysis has revealed a peculiar cancer-associated ECM structure [121]. In this study, ECM stiffness is favored by the overexpression of collagen type III alpha 1 chain (COL3A1) as a component of tumor-associated aligned collagen that promotes iCCA cell migration. Furthermore, the desmoplastic nature of iCCA displays a mesenchymal component that prevails over the epithelial counterpart, with a large proportion of cancer cells expressing EMT traits. This functional dynamic program appears to play an active part in iCCA tumorigenesis related to tumor progression, prognosis, and metastatic potential. This latter is facilitated by enhanced migratory and invasive properties of cancer cells due to disruption of cell–cell adhesion, cellular polarity, remodeling of the cytoskeleton, and changes in cell–matrix adhesion [213,214,215]. Indeed, accurate mass and time tag proteomics (AMT) in iCCA tumor cells highlighted an altered protein network involved in cytoskeleton organization and cell motility. In particular, a striking increase in vimentin and actin-binding proteins (i.e., cofilin-1, profilin-1, and transgelin-2) was observed, suggesting their potential role as markers in iCCA progression [122]. Interestingly, proteomics analysis revealed a decrement in the cell–cell junction level in iCCA patients with HBV infection (HBV-iCCA). Indeed, based on the deconvolution method “xCell” integrated with the proteomics dataset, an inverse relationship between the cell–cell junction level and the EMT program has been demonstrated. In this study, it has been noted that the epithelial cell abundance is lower in HBV-iCCA rather than in non-HBV-iCCA patients. Through correlation analysis, the lower number of epithelial cells is related to the E-cadherin reduction, suggesting an increment of the EMT program in HBV-iCCA patients [123].

Deep differences between iCCA and HCC in terms of epidemiology, diagnosis, outcome, and therapies highlight the unique aggressive nature of the iCCA [216,217].

Using the data-independent acquisition mass spectrometry (DIA-MS), a recent study showed that these two primary liver cancers differ in their proteomics landscape [124]. The molecular signature that discriminates between the two forms of liver cancer is the expression of proteins involved in lipid metabolism, such as alpha-methylacyl-CoA racemase (AMACR), sterol regulatory element-binding proteins (SREBP), and fatty acid synthase (FASN), that were found enriched in HCC tumors. Furthermore, inhibition of pathways related to oxidative phosphorylation, serotonin degradation, valine degradation I, and xenobiotic metabolism has been observed in iCCA compared to HCC.

The authors also demonstrated that iCCA is characterized by an enrichment of ECM-related pathways (i.e., laminin interactions, assembly of collagen fibrils, PTK2 signaling), thus highlighting a particular pathogenic predisposition to developing tumor metastasis and a worse prognosis than HCC. In particular, upregulation of S100A6 (S100 calcium-binding protein A6), a protein that plays an important role in the reorganization of the cytoskeleton and cellular motility, has been detected in the iCCA proteome. In this regard, the authors suggest S100A6 as a useful and potential biomarker to distinguish the two forms of primary liver cancer.

It is known that iCCA spreads mainly via the lymphatic system due to the predominant sprouting of lymphatic vessels over the blood vasculature. Indeed, patients with high lymphatic microvessel density (LVD) have an increment of nodal spread and frequently develop recurrence [218]. Hence, knowledge of the molecular events underlying the iCCA hypovascularity and neo-lymphangiogenesis is another important step in identifying molecular targets for therapeutic interventions to counteract the invasiveness of iCCA. Analyzing the proteomic landscape of iCCA-ECF obtained by iCCA patients, we unveiled the presence of an angio-inhibitory milieu, characterized by overexpression of thrombospondin 1 and 2 (THBS1, THBS2) and pigment epithelium-derived factor (PEDF) that hinder the vascular network and promote the trans-differentiation of vascular endothelial cells toward a lymphatic phenotype. Most importantly, in mouse xenograft experiments with CCLP1, an established human cell line of iCCA, we demonstrated that the simultaneous presence of blocking antibodies for THBS1, THBS2, and PEDF in the iCCA-ECF was able to drastically reduce the tumor growth and the extent of tumor infiltration in local lymph nodes [40].

### 4.2. Metabolomics

Metabolomics provides a chemical fingerprint of metabolism in healthy cells and changes in this pattern can reveal disease states related to inflammation or cancer processes. This is particularly useful in the context of iCCA [201,219].

It is known that metabolic reprogramming is a distinctive feature of cancer cells causing their malignant behavior and their survival under resource-limited conditions by exploiting alternative nutrient pathways. In this scenario, even in iCCA pathobiology, an altered glucose metabolism (Warburg Effect) along with a higher mitochondrial activity compared to normal cholangiocytes plays a predominant role in iCCA tumor cancer cells [220].

It has been assumed that the involvement of an altered and increased glycolysis in iCCA due to a greater expression of fructose-bisphosphate aldolase A, lactate-dehydrogenase A, and glucose transporters indicates that cancer cells tend to favor glycolysis to maintain their faster growth rate [122]. Related to this, a recent quantitative metabolomic study demonstrated marked differences in terms of glycolysis and gluconeogenesis rate between LDT and SDT [125]. A marked downregulation in the expression of triosephosphate isomerase 1 (TPI1), glyceraldehyde-3-phosphate dehydrogenase (GAPDH), and phosphoglycerate kinase 1 (PGK1) was observed in LDT compared to SDT, thus providing a novel potential metabolomic signature for the pathological classification of iCCA patients. Additionally, a significant correlation between altered glycolysis and EMT has been demonstrated, due to an upregulation of the facilitated SLC2A3 (glucose transporter member 3) in iCCA cells. This elucidates the intricate interplay between metabolic reprogramming and the aggressiveness of this biliary tumor [126].

In a small cohort of Italian iCCA patients, an upregulation of components related to oxidative metabolism has been found, causing the accumulation of free radicals [127]. Indeed, overexpression of catalase, peroxiredoxin 2 and 6 (PRDX2, PRDX6), and superoxide dismutase (SODM) have been found in iCCA tissue compared with the normal adjacent one.

It is known that intrahepatic bile duct stones (IBDS) are one of the most common independent risk factors that cause iCCA occurrence [221]. Using the gas chromatography–mass spectrometry (GC-MS) approach, a deep difference has been demonstrated between IBDS and iCCA patients in terms of the linoleic acid metabolism pathway. Specifically, the amount of 9,12-octadecadienoic acid was found to be significantly higher in IBDS patients compared to iCCA patients, suggesting its potential use as a marker for long-term monitoring and symptomatic treatment of IBDS and, consequently, to prevent the iCCA incident [128].

Regarding useful strategies for the non-invasive and early diagnosis of iCCA, Haznadar and co-workers demonstrated the valuable efficacy of the metabolites detected in the urine of iCCA patients [129]. Specifically, four urinary metabolites, creatine riboside (CR), N-acetylneuraminic acid (NANA), cortisol sulfate (CS), and a lipid molecule designated as 561+, were found to be more abundant in urine collected from iCCA patients compared to those with HCC. Notably, high levels of CR were correlated with poorer diagnosis. Furthermore, the authors suggest that the combination of these four metabolites with the presence of CA19-9 increases iCCA diagnostic potential in terms of sensitivity and specificity [129].

## 5. Multi-Omics Approaches in iCCA: A New Model of Knowledge

As mentioned above, iCCA is classified into two main subtypes, SDT and LDT, based on histology, location, metabolic profile, and key mutations [30,31,32,222]. Each of these subtypes exhibits high inter- and intratumor heterogeneities. Therefore, the SDT and LDT categories are not exhaustive for iCCA taxonomy, making the search for early diagnostic tools and effective therapeutic strategies a real and complex challenge [14,33]. Single-omics approaches shed light onto this intricate heterogeneity, but recently researchers have moved toward a multidimensional study through multi-omics methods to gain a more comprehensive understanding of iCCA for better patient stratification (Table 2). In this paragraph, we will report the initial steps toward this new multi-omics model of iCCA.

The first attempts were performed in 2017 by integrating genomic and epigenomic data [88,189]. In these initial studies, Jusakul and colleagues divided iCCA patients into four cluster groups, unveiling a close correlation between phenotype and etiological agents. Cluster 1 and 2 are driven by external carcinogenic agents, epimutations, and *ARID1A*, *BRCA1/2*, and *TP53* mutations. Specifically, cluster 1, detected as liver fluke-positive, differs from cluster 2 in the hypermethylation rate of CGIs. Clusters 3 and 4 are both liver fluke-negative and were characterized based on immune response and genomics characteristics. Cluster 4 was specifically represented by patients with *IDH1/2* mutations, *FGFR2* fusion, and HBV infection [189]. Farshidfar and colleagues, instead, performed a multi-omics analysis on a cohort of 38 iCCA patients specifically selected as liver fluke-negative [88]. They stratified the patients into distinct groups according to somatic mutations, RNA expression, CNAs, and DNA methylation. Data from TCGA, supplemented with multiplatform analyses, identified a specific iCCA subtype with *IDH1/2* mutations. Furthermore, it has been demonstrated that this subtype shows upregulation of mitochondrial genes and hypermethylation of the promoter CpG region, resulting in low expression of chromatin modifier genes such as *ARID1A* and *ARID1B* (AT-rich interaction domain 1B).

In 2019, Goeppert and collaborators [97] performed genomic and epigenomic characterization of 52 iCCA tissues. Based on the CGI methylation rate, distinct clusters were detected. Specifically, general hypermethylation was observed in iCCA samples: 37,600 CGIs showed increased methylation of more than 20%, while only 2217 CpG exhibited hypomethylation of less than 20%. The hypermethylated regions were enriched in transcription start sites and enhancers; on the contrary, the hypomethylated were enriched in coding sequences and heterochromatic regions. Using genetic and epigenetic integrative clustering, they identify four iCCA subgroups named the IDH group (patients with *IDH1* and *IDH2* mutations), L (low alteration) group, M (medium alteration) group, and H (high alteration) group, each one showing specific features. Methylation patterns were different between L and M vs. H and IDH: general hypermethylation was confirmed in all groups, but the IDH and H groups showed broad hypermethylation with highly group-specific patterns, while only a few CGIs with group-specific hypermethylation were reported in the M group using the L group as reference. Some genes considered cancer drivers (*ROBO2*, *RPL22*, *ROBO1*, and *TGFBR1*) were altered in all groups because of mutations or promoter hypermethylation. These four subgroups showed different outcomes: the M group had the worst OS, followed by the H group, while the L and IDH groups showed the best prognosis.

As previously mentioned, Liao et al. identified four subtypes of iCCA based on the epigenomics approach [102]. They found specific epigenetic characteristics associated with OS. Subsequently, the authors applied a multi-omics approach using data on DNA methylation and expression at both the RNA and protein levels and showed that the variable PMDs affected gene expression involved in iCCA carcinogenesis. In the context of the general hypomethylation observed across iCCA subgroups, the most hyper-differentially methylated regions (DMRs) were observed in S1, S3, and S4, while S2 was enriched in hypo-DMRs. Hyper-DMRs were enriched in CGIs, confirming the focal hypermethylation of iCCA in CGIs; hypo-DMRs were instead enriched in H3K27ac-associated regions and transcriptional binding sites. Overall, a positive association between averaged global and CGI methylation levels was reported across iCCAs.

Many aspects of the intratumor heterogeneity are closely related to the immune microenvironment, which allows for the identification of different tumor subtypes [233,234,235]. The immunological heterogeneity of the tumors helps to diversify patients based on immune profile, and this stratification is useful for specific PM based on the advanced tumor-targeting immunotherapy [236]. Although the immune microenvironment landscape of iCCA is not fully understood, the importance of the inflammatory state related to tumor progression and cancer cell dissemination through the regulation of TME components is well established [237,238].

One of the first classifications focused on the immune microenvironment was carried out by Sia and colleagues who combined genomic data with mutational analyses to identify two classes of iCCA: “inflammation” and “proliferation” [95]. The inflammation group is characterized by the activation of inflammatory signaling pathways, overexpression of IL-4 and IL-10 cytokines, and STAT3 activation. In contrast, the proliferation group shows activation of oncogenic signaling pathways, including MAPK, DNA amplifications at 11q13.2, deletions at 14q22.1, mutations in *KRAS* and *BRAF*, and gene expression signatures previously associated with poor outcomes for patients with HCC. The proliferation class presents a worse prognosis than the inflammation class [95,239].

In another study, Job and colleagues [103] showed that iCCA TME displays different escape mechanisms from immune response, grouped into four subtypes: immune desert, immunogenic, myeloid, and mesenchymal. The immune desert subtype is the most widespread among the cases analyzed, characterized by the depletion of both MHC class I and T cells. The immunogenic subtype, instead, presents an inflammatory phenotype by including many immune components surrounding cancer cells. This subtype results in a highly inflamed TME counteracted by the activation of inflammatory and immune checkpoint pathways, and quiescent hepatic stellate cells (HSC), known to have a key role in transdifferentiating into matrix-producing myofibroblast in liver fibrogenesis [240]. In addition, it is associated with the longest patient survival and is considered a potential target for Immune Checkpoint Inhibitors (ICI) therapies. The myeloid subtype is characterized by the infiltration of macrophages M2 and CD4^+^ T lymphocytes. The massive abundance of M2 macrophages sustains their anti-inflammatory and immunosuppressive activities, which could explain the deficiency of adaptive immune response in this subcategory. In addition, some of the patients of the myeloid subtype are positive for hepatitis C virus (HCV) infection, which is known to prompt monocytes to differentiate into the M2 macrophage. Lastly, the mesenchymal subtype displays high levels of vascular factors, chemokines, and paracrine factors produced by CAFs, which enhance pro-tumorigenic pathways and restrain immune cell recruitment into the tumor tissue.

Recently, a combination of proteomics, whole-exome sequencing (WES), and scRNA-seq has brought to light three molecular subtypes of iCCA classified in chromatin remodeling, metabolism, and chronic inflammation [223]. It has been observed that the chronic inflammation subtype is associated with poor prognosis. Particularly, chronic inflammation has a large population of APOE^+^C1QB^+^ tumor-associated macrophages (TAMs) that promote the secretion of TNFα by the CD4^+^ T lymphocytes contributing to the development of the inflammatory state. This work is consistent with the study conducted by Job and colleagues highlighting how macrophage and other immune cell populations are important in reshaping TME in iCCA. On the contrary, in the metabolism subtype, the mutation of lysine methyltransferase 2D (*KMT2D*) is significantly involved in the inflammatory response and metabolic activity of iCCA, and is associated with a better prognosis.

Another kind of subdivision has been carried out on tumor-associated tertiary lymphoid structures (TLSs) that represent an important milieu for both cellular and humoral immune responses against cancer. Commonly, TLS intratumoral detection is correlated with favorable prognosis and response to immunotherapy in many solid tumors [234,241].

Although the prognostic and clinical value of TLSs in the iCCA context is still lacking, a recent study describes the TLS spatial distribution and abundance in specific anatomical subregions (intra- and peri-tumoral) of iCCA patients [224]. The authors reported that TLSs in intratumoral regions (T) were found associated with a favorable prognosis for the patients, unlike the TLSs residing in peri-tumoral regions (P) correlated to dismal outcomes. Considering this distribution, a combination of T and P scores was used. These two parameters allowed the identification of four immune subclasses with significantly different prognoses. These classes reflect the spatial distribution, abundance, and functional orientation of TLSs in iCCA patients. Specifically, class I (immune excluded) has minimal intratumoral TLSs and massive peri-tumoral TLSs, and it is associated with a worst prognosis. Conversely, class IV is characterized by an abundance of TLSs in intratumoral regions rather than in the peri-tumoral, reporting a strong and effective anti-tumor immune response (immune active). In class II and III, there is an intermediate distribution pattern of TLSs, reflecting an altered immune balance, which is directed to either pro-tumor or anti-tumor orientations (immune altered).

By integrating multimodule data based on genomics, transcriptomics, and proteomics, Lin and colleagues defined three immune subgroups (IG1, IG2, IG3) of iCCA [225]. In IG1, the highest myeloid infiltration and *KRAS* mutation rate has been detected, resulting in an immune-suppressed phenotype and the worst prognosis. On the contrary, IG2 is characterized by tumor proliferation and antigen presentation defects, while IG3 presents antitumor immunity and T enrichment of TLSs with a good prognosis. Moreover, IG1 could be responsive for myeloid-targeted therapies as those based on CXCR2 (C-X-C motif chemokine receptor 2) and CSFR (colony-stimulating factor receptor) inhibitors to deplete or reprogram tumor-associated neutrophils and M2 macrophages. In agreement with Ding’s study [224], Lin et al. revealed that the enrichment of TLSs in IG3 could have a potential role in the response to ICI treatment [225].

In another report, through a multi-level analysis with WGS, RNA-seq, and mIHC on 207 tumor regions from 45 patients, Lin and collaborators portrayed the immunogenomic landscape of iCCA [226]. They delineated a mutual dynamic correspondence between immune composition and function and iCCA’s genetic makeup. In particular, the authors highlight how immune heterogeneity affects the tumor development of iCCA. Indeed, the 45 iCCA patients were subclassified into the so-called immune groups: sparsely infiltrated, heterogeneously infiltrated, and highly infiltrated. Immunogenomic analysis revealed that the sparsely infiltrated group exhibits an inadequate number of chemokines with low infiltration of immune and stromal cells. The heterogeneously infiltrated group shows a distinct distribution of chemokines between tumor subregions with low and high immune infiltrations. The sparse and heterogeneous groups present comparable levels of both TNB (tumor neoantigen burden) and TMB (tumor mutation burden). On the other hand, the highly infiltrated group displays the highest TNB and the lowest tumor purity, abundant immune infiltration, and overexpression of the immune checkpoint. Concerning the transcriptomics profile, the sparsely infiltrated group showed an upregulation of cell-cycle signaling, oxidative phosphorylation, and cholesterol homeostasis. Conversely, the highly infiltrated tumors were characterized by the overactivation of immune-related mechanisms, angiogenesis, and cancer pathways. Overall, based on these specific characteristics, the authors suggest that immune infiltration alone is not sufficient to distinguish the outcome of the iCCA patients, thus suggesting a further prognostic classification based on the immune evasion ability.

Using mIHC analysis, it was demonstrated that the spatial immunophenotypes can predict the clinical outcome in iCCA [227]. In this study, the multiregional immune context of 192 treatment-naïve iCCA patients was evaluated through the quantitative analysis of 16 immune cell subsets in distinct areas: peritumor, invasive margin (IM), and intratumor. From these analyses, immune cell subsets have a region-specific distribution in tumor tissue. In particular, the intratumor area displays scarce infiltration of most immune cell subsets except for CD15+ neutrophils. Based on these observations, each area was subdivided into immune-high and immune-low subgroups leading to the identification of three spatial immunophenotypes: inflamed, excluded, and ignored. According to Ding et al. [224], the inflamed phenotype shows a significant abundance of immune cell infiltration in the intratumor area, while the ignored and excluded phenotypes constitute the non-inflamed phenotypes. The ignored phenotype shows immune-low features in all three areas while the excluded phenotype presents a low number of immune cell infiltration confined to the IM and peritumor areas. Subsequently, the authors revealed that these three mentioned immunophenotypes have distinctive molecular features and different OS. While the inflamed phenotype shows overexpression of PDL-1 (programmed cell death 1 ligand 1) and relatively favorable OS, the excluded phenotype, characterized by moderate prognosis, has an upregulation of activated HSCs, extracellular matrix, and Notch signaling pathways. Lastly, the ignored phenotype is associated with poor prognosis and shows an up-stimulation of MAPK signaling. Furthermore, the non-inflamed phenotypes share increased angiogenesis, the upregulation of TGFβ and WNT-beta catenin pathways, and are rich in *BAP1* mutations and *FGFR2* fusions. In addition, according to immune escape mechanisms, the authors suggest that iCCA patients with the inflamed phenotype may be potential target populations for traditional ICI based on PDL-1 or CTLA-4 (cytotoxic T-lymphocyte protein 4). On the other hand, patients with non-inflammatory phenotype, due to the low number of cell infiltrates CD8^+^ T, may undergo novel immunotherapies using CD276 (CD276 molecule) or VTCN1 (V-set domain-containing T-cell activation inhibitor 1).

Another molecular classification of iCCA has been proposed considering the characteristics related to the stroma, tumor, and immune microenvironment, the so-called STIM classification [228]. This has led to the development of five STIM classes. The first two, immune classical and inflammatory stroma, concern the inflammatory profiles, both characterized by high immune infiltration and extensive desmoplasia. In particular, the immune classical is marked by cell-cycle pathways and poor prognosis. In contrast, the remaining three categories have been divided into non-inflammatory, desert-like, and hepatic stem-like classes, characterized by low immune infiltration.

The iCCA heterogeneity may also be determined by the B cell population level detected within the TME of patients [242]. Indeed, once lymphocyte tumor infiltration occurs, there is a higher frequency of naïve rather than memory B cells, which create cellular aggregates like TLSs. Additionally, B cell heterogeneity is also found in plasma. iCCA patients who responded to the treatment with gem-cis plus durvalumab showed a higher frequency and more activated naïve B cells compared to the non-responder who presented an increase in memory B cells.

Based on the median inter-tumor heterogeneity (ITH) index, another recent report conducted on 66 tumor samples from 16 patients with multifocal iCCA divided patients into high- and low-ITH groups [229]. Patients with multiple lesions displayed marked genomic, transcriptional, and epigenetic tumor heterogeneities in the ITH group. This group also showed a strong epigenetic component, characterized by a marked prevalence of driver gene mutations associated with epigenetic modifiers and mutations in genes related to the Wnt and TGFβ pathways. This implies that tumors with a high ITH index are more aggressive than those with a low ITH index. Subsequently, using CD8B (CD8 subunit beta) and ICOS (inducible T cell costimulator), iCCA patients were classified into two immune clusters, the high-immune and low-immune, consistent with the above ITH-clustering. Both clusters reveal a strong correlation between OS and RFS. Indeed, the high-immune cluster showed high expression levels of *CD8B* and *ICOS* genes, and it was associated with better prognosis rather than the low-immune cluster, suggesting *CD8B* and *ICOS* genes as potential prognostic markers and direct targets for personalized immunotherapy.

Integrated multi-omics analysis performed on 102 iCCA patients identified three clinically supported subtypes with specific molecular features and different OS (i.e., “stem-like”, “poorly immunogenic”, and “metabolism”) [231]. The metabolism group, characterized by *IDH1* and *BAP1* mutations, showed a good prognosis. Conversely, the poorly immunogenic subtype, characterized by a lower number of CD3B and CD8B cells as well as by high frequencies of *KRAS* and *TP53* mutations, showed the worst prognosis. The stem-like subtype, with a high frequency of *ALDH1A1* mutations, showed an intermediate prognostic value with respect to those observed for the metabolism and poorly immunogenic subtypes. Overall, this large-scale analysis supports the importance of iCCA patient stratification to develop tailored medicine strategies.

Dong and colleagues performed an integrative genomic, transcriptomic, proteomic, phosphoproteomic, and microbiome analysis of 262 Chinese iCCA patients [230]. This complex proteogenomic analysis identified four different subgroups (S1–S4). Each subgroup has different features in terms of genetic alterations, microenvironment dysregulation, tumor microbiota composition, and OS. Correlation analysis based on multi-omics data revealed two potential prognostic protein biomarkers, the HKDC1 (hexokinase domain containing 1) and the SLC16A3 (monocarboxylate transporter 4). Some tumors display high HKDC1 expression levels, which negatively correlated with factors involved in central carbon metabolism, IL-17 signaling, cytoskeleton regulation/ECM interaction, and PI3K-AKT pathways. On the other hand, patients with high SLC16A3 expression showed increased levels of proteins involved in the active carbon metabolism, cytoskeleton regulation/ECM interaction, and complement signaling pathways, but lower steroid hormone biosynthesis and drug metabolism pathways. Thus, considering that inflammation and stromal responses promote the iCCA progression, HKDC1 may act as an iCCA suppressor, while SLC16A3 could affect the recruitment of immune cells promoting tumor growth.

Multi-omics analyses have revealed metabolomic subtypes of iCCA patients [232]. Analyzing 116 iCCA samples, the authors describe three metabolic profiles (S1–S3). Among them, the S2 subtype showed the worst survival outcome and is characterized by high frequencies of *KRAS/ARID1A* mutations, enrichment in NAD(P)^+^, downregulation of enzymes involved in fatty acid degradation, and upregulation of enzymes involved in glycolysis. According to previous studies [243], the authors suggest that *KRAS* mutations could upregulate the activity of key glycolytic enzymes to promote glycolysis and utilize fatty acid synthesis to regenerate NADP^+^, all metabolic features of the iCCA cells.

The stratification of iCCA patients obtained through multi-omics approaches is driven by advances in algorithmic capabilities, machine learning, and artificial intelligence (AI), which are increasingly employed in cancer research to develop prognostic models. This progress capitalized on data from various omics platforms enabling multi-level analyses that not only create prognostic models but also tailor therapies to individual patient phenotypes. Notably, a recent editorial emphasizes that AI and multi-omics methods could represent the future of iCCA prediction research [244]. Specifically, it is hypothesized that integrating advanced image processing such as radiomics, and pathomics with multi-omics analysis and AI algorithms could lead to the development of “super models” with significant prognostic potential for iCCA.

## 6. Conclusions

In this review, we aim to present a cutting-edge perspective of the iCCA through the lens of omics approaches. Each of the multi-omics approaches discussed in this review proposes the stratification of iCCA patients into different subtypes based on the molecular features observed in individuals. These classifications aimed to pave the way to a PM for enhancing diagnosis and therapeutic intervention. The studies associate each subtype with either an adverse or favorable prognostic impact, therefore suggesting a link between molecular characteristics and prognosis.

**Figure 2:**
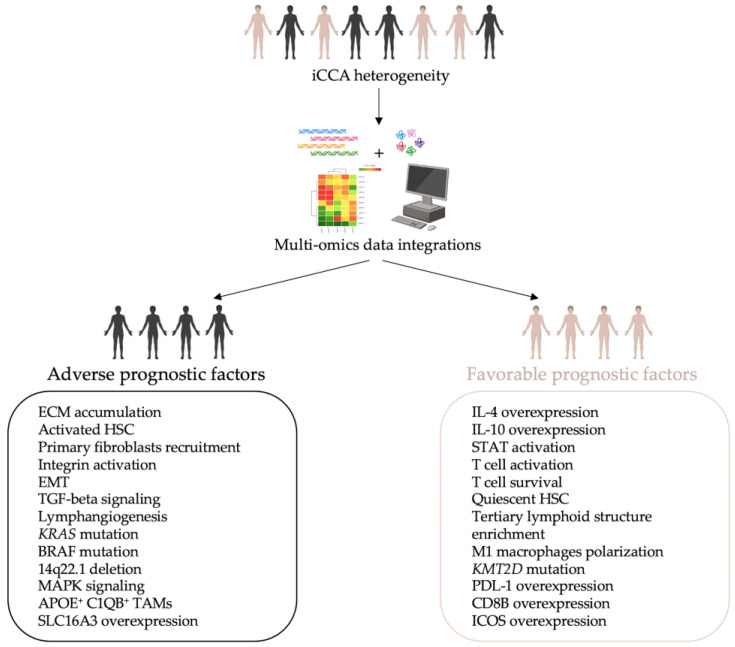
Prognostic biomarker lists for iCCA based on the multi-omics data. Cellular and molecular factors identified as prognostically relevant in multi-omics studies cited in this review were categorized as “adverse” or “favorable” according to their prognostic impact. Inter-tumor heterogeneity of iCCA patients is indicated with people with different colors.

Based on the multi-omics studies reviewed, we propose a tentative list of biomarkers and molecular pathways that emerged, categorized into two clusters: adverse prognostic factors and favorable prognostic factors (Figure 2). The adverse prognostic factors, associated with a worse prognosis, include the development of a reactive desmoplastic stroma arising from the mesenchymal cellular component of the TME. This involves primary fibroblast recruitment, ECM accumulation, integrin activation, lymphangiogenesis, EMT, and TGF-beta signaling. Among these, primary fibroblast recruitment, *KRAS* mutation, and lymph-nodes metastasis via neo-lymphangiogenesis have been demonstrated to be accurate and reliable adverse prognostic factors by long-term follow-up analysis [154,245,246]. In contrast, the favorable prognostic factors, associated with a better prognosis, are characterized by a high immune response, such as T cell activation and survival, overexpression of CD8B and PDL-1, and TLS enrichment. Notably, in the frame of TLS enrichment in iCCA patients, displaying an immune-active pattern leads to a favorable prognosis, with 5-year OS rates of 68.8% [224].

Further studies are required to refine this classification system to address the challenges of early diagnosis and to improve the prognosis of iCCA, which unfortunately remains poor worldwide.

## Figures and Tables

**Table 1:** Single-Omics approaches in iCCA.

Single-Omics	Sample Type	Methods	Results	Refs.
**Genomics**	Ex vivo	RNA-seq; FISH; WES; Gene-array	Identification of 13 novel inter- and intrachromosomal fusion events; Top ranked fusion event: fusion of *FGFR2* and *PPHLN1*	[89]
	Ex vivo	NGS	5547 genomic alterations in 335 genes: 3424 short variants, 1676 copy number variations	[43]
	Ex vivo	WES	Detection of fusion transcripts *FGFR2-AHCYL1*, *FGFR2-* *BICC1*, *AHCYL1-FGFR2*, and *BICC1-FGFR2*	[90]
	Ex vivo	NGS	Most commonly mutated genes: *KRAS* (28.1%), *TP53* (18.3%), *ARID1A* (11.8%), *IDH1/IDH2* (9.2%), *PBRM1* (9.2%), *BAP1* (7.2%), and *PIK3CA* (7.2%)	[91]
	Ex vivo	NGS	1259 somatic mutations in 1128 genes	[92]
	Ex vivo	Gene-array	775 genetic alterations and 38 structural alterations	[42]
	Ex vivo	NGS, WES, and WGS	Most recurrently mutated genes: *TP53*, *KRAS*, *IDH1/2*, *ARID1A*, *BAP1*, *PBRM1*, *FGFR2* fusion, *CDKN2A*, *SMAD4*, *PIK3CA*, *EPHA2* (mutation frequency > 5%)	[93]
	Ex vivo	NGS	Most commonly mutated genes: *TP53*, *ARID1A*, *KRAS*, *CDKN2A*, *SMAD4*, and *PBRM1*	[94]
	Ex vivo	SNP-array	Copy number gain: chromosome 1q (32%) and 7p (25%); Copy number loss: 6q (52%), 9q (45%), 9p (42%), 3p (41%), 13q (38%), 14q (36%), 8p (30%), 21q (20%), 17p (21%), 4q (18%), 1p (16%), 18q (15%), and 11p (13%)	[95]
	Ex vivo	Exome-array	Exclusively deleted cytobands: 1p36.33–1p35.1, 3p26.3–3p14.25, and 14q24.1–14q32.33; Exclusively amplified cytobands: 1p11.2–1p41.1, 1q21.1–1q44, 2q23.1–2q35, 7p22.3–7p11.1, 7q11.1–7q36.3, and 8q23.2–8q24.3	[96]
**Epigenomics**	Ex vivo	Methylation-array	Methylation data used to classify tumors; general hypermethylation in iCCA samples	[97]
	Ex vivo	Methylation-array	cg15026696 hypomethylation and cg06972969 hypermethylation	[98]
	In vitro	ChIP-sequencing	Reduced levels of H3K4me1, H3K4me3, and H3K27ac in tumor cells compared to normal cells	[99]
	Ex vivo	Methylation-array	2048 most variable CpG sites used to classify tumors	[100]
	Ex vivo	WGBS	Identification of prognostically methylated regions (PMRs) as biomarkers	[101]
	Ex vivo	WGBS	DNA methylation data used to classify tumors	[102]
**Transcriptomics**	Ex vivo	Transcriptome-array	Gene expression profiles used to classify iCCA tumors	[103]
	Ex vivo	RNA-sequencing	Gene expression profiles used to classify iCCA tumors	[104]
	Ex vivo	RNA-sequencing and Transcriptome-array	*C19orf33*, *S100P*, *CEACAM6*, *KRT17*, *GPRC5A*, *MSLN*, *SERPINB5*, *TCN1*, *OLFM4*, *VTN*, *ADH1C*, *ALDOB*, *FABP1*, *ADH1B*, *UGT2B7*, *APOC4*, *AQP9*, *CDO1*, *GSTA2* as biomarkers of specific iCCA subgroups	[105]
	Ex vivo	mRNA and microRNA-array	2327 DE mRNAs (1113 up- and 1214 downregulated) and 70 DE miRNAs (65 up- and 5 downregulated) in iCCA vs. paired non-cancerous liver tissue samples	[106]
	Ex vivo	RNA-seq	230 DE lncRNAs (97 up- and 133 downregulated) and 2220 DE mRNAs (640 down- and 1580 upregulated) in iCCA vs. paired unaffected tissues; top downregulated genes: *RP11-328K4.1*, *LINC01093*, *LINC00844*, *RP11-372E1.4*, *ITIH4-AS1*; top upregulated genes: *RP11-532F12.5*, *AC016735.1*, *RP11-284F21.7*, *LINC01123*, *AC013275.2*	[107]
	Ex vivo	GEP; Transcriptome-array; NGS	315 DE genes (65 down- and 250 upregulated) in recurrent compared with primary tumors	[108]
	Ex vivo	miRNA-seq	miR-10b, miR-22, and miR-551b significantly associated with OS in iCCA patients; Overexpression of miR-551b decreased HuCCT-1 proliferation and promoted apoptosis	[109]
	Ex vivo	RNA-sequencing	1643 DE transcripts (1098 up- and 545 downregulated)	[110]
	Ex vivo	microRNA-array, and RNA-sequencing	Pathway enrichment analysis performed on DE lncRNAs and mRNAs revealed their involvement in the Hippo pathway	[111]
	Ex vivo	Transcriptome-array	Gene expression profiles used to classify iCCA tumors; identification of ICCA-specific DE genes (395 up- and 5889 downregulated); Upregulated: *ARHGAP21*; Downregulated: *SCP2*, *UBIAD1*, *TJP2*, *RAP1A*, *HDAC9*, *FKBP2*, *MRPL2*, and *MRPL27*	[112]
	Ex vivo	Transcriptome-array	Classification of iCCA tumors according to 794 DE transcripts (486 up- and 308 downregulated) specific for cholangiolocellular differentiation (CD); specific of CD-tumors: CRP; specific of non-CD-tumors: *S100P*, *TFF1*, *AGR2*, *CLDN18*, *KRT17*, and *CTSE*	[113]
	Ex vivo	single-cell RNA-seq	Gene expression profiles used to classify cells	[114]
	Ex vivo	RNA-sequencing	Gene expression profiles used to classify iCCA tumors	[115]
	Ex vivo	Transcriptome-array	2611 (1142 up- and 1469 downregulated)	[116]
	Ex vivo	STS; WES; WGS; NGS; Methylation-array	Gene expression profiles used to classify iCCA tumors; 52 genes showed differential expression between Cluster1A and Cluster2	[93]
	Ex vivo	circRNA-sequencing	4 circRNAs upregulated in metastatic vs. non-metastatic tumors (*circZNF215*, *circBNIP3L*, *circCD109*, and *circPLOD2*)	[117]
	Ex vivo	Transcriptome-array	173 DE circRNAs (69 up- and 104 downregulated), 58 DE miRNAs (30 up- and 28 downregulated), and 4437 DE mRNAs (2234 up- and 2203 downregulated)	[118]
	Ex vivo	RNA-sequencing	262 dysregulated miRNAs (128 up- and 134 downregulated); top upregulated miRNAs: miR-21, miR-34c, miR-200b, and miR-221	[119]
**Epitransctriptomics**	Ex vivo	Methylated RNA immunoprecipitation sequencing	*METTL14 p.R298H* mutation affected m6A modifications on the target *MACF1*	[120]
**Proteomics**	Ex vivo	Nano-LC-MALDI-TOF/TOF	Overexpression of COL3A1 and low levels of reticular and elastic fibers; Basement membrane dismantling; Reduced angiogenesis; COL3A1 promotes iCCA cell migration	[121]
	Ex vivo	AMT Tag	Altered protein network involved in cytoskeleton organization and cell motility (increase in vimentin, cofilin-1, profilin-1, and transgelin-2, S100A11, reduction in annexins)	[122]
	Ex vivo	TMT	Decreased cell–cell junction level in HBV-iCCA; Inverse relationship between cell–cell junction level and EMT program: reduced abundance of epithelial cells is correlated with low expression levels of E-CAD in HBV-iCCA	[123]
	Ex vivo	DIA-MS	Enrichment of proteins related to lipid metabolism (i.e., AMACR, SREBP, FASN) in HCC tissues; High levels of proteins involved in ECM-related pathways (S100A6, laminin interactions, assembly of collagen fibrils, PTK2 signaling) in iCCA tissues; S100A6 potential diagnosis biomarker to discriminate iCCA from HCC.	[124]
	Ex vivo	LC-MS/MS	Overexpression of angio-inhibitory proteins in iCCA-ECF; Upregulation of THBS1, THBS2, and PEDF inhibits angiogenesis on behalf of lymphangiogenesis promoting the trans-differentiation of vascular endothelial cells toward a lymphatic phenotype	[40]
**Metabolomics**	Ex vivo	iTRAQ labeling and LC-MS/MS	Downregulation of enzymes (TPI1, GAPDH, and PGK1) involved in glycolysis and gluconeogenesis pathways in LDT compared to SDT	[125]
	Ex vivo	MS	High mitochondrial activity, with an enrichment of glutamine and glucose uptake; upregulation of glucose transporter SLC2A3; positive correlation between SLC2A3 and EMT markers (i.e., N-CAD)	[126]
	Ex vivo	MALDI-MS and MALDI-MS/MS	Upregulation of enzymes (i.e., CAT, PRDX2/6, and SODM) involved in redox homeostasis causing accumulation of free radicals in iCCA patients	[127]
	Ex vivo	GC–MS	Overactivation of linoleic acid metabolism pathway in IBDS rather than in iCCA tissues; 9,12-octadecadienoic acid potential biomarker for long-term monitoring, and treatment of IBDS to prevent the risk of iCCA development	[128]
	Ex vivo	UPLC-MS/MS	4 urinary metabolites (CR, NANA, CS, and 561+) are upregulated in iCCA compared to HCC patients; Potential model of iCCA diagnosis based on the combination of the 4 urinary metabolites and CA19-9	[129]

**Table 2:** Multi-Omics analyses in iCCA.

Multi-Omics	Sample Type	No of iCCA Patients	Methods	Results	Refs.
Genomics + Epigenomics	Ex vivo and In vitro	71	WGS; MiGS	4 clusters based on strict correlation between phenotype and etiological agents	[189]
Genomics + Epigenomics + Transcriptomics	Ex vivo	38	NGS; WES; RNA-seq; miRNA-seq; SNP-array	4 clusters according to genomics, epigenomics, and transcriptomics profile	[88]
Genomics + Epigenomics	Ex vivo	52	WGS; WES	4 subgroups (IDH, L, M, H) based on genetic and epigenetic features	[97]
Genomics + Epigenomics integrated with WES, RNA-seq, and proteome datasets	Ex vivo and Invitro	331	WGBS; TMA; WES	4 clusters (S1–S4) according to hyper-differentially methylated regions (DMRs)	[102]
Genomics + Epigenomics + Transcriptomics	Ex vivo	149	bs-DNA; Methylation-array; RNA-seq	2 iCCA classes (inflammation and proliferation) based on genomic and mutational profiles	[95]
Epigenomics + Transcriptomics	Ex vivo	116	Methylation-Array; Transcriptome-Array	4 iCCA subtypes are identified according to different escape mechanisms in TME (immune desert, immunogenic, myeloid, and mesenchymal	[103]
Proteomics + Genomics + Epigenomics + Transcriptomics	Ex vivo	110	TMT; NGS; WES; Single-cell RNA-seq	3 molecular iCCA subtypes (chromatin remodeling, metabolism, and chronic inflammation) correlated to OS	[223]
Proteomics + Epigenomics	Ex vivo	962	mIHC; WES	4 iCCA classes based on the spatial distribution, abundance, and functional orientation of TLSs (immune excluded, altered, active)	[224]
Genomics + Transcriptomics + Proteomics	Ex vivo	255	WES; Single-cell RNA-seq; MS; mIHC + TMA;	3 iCCA subgroups (IG1-3) established by immune landscape	[225]
Genomics + Transcriptomics + Proteomics	Ex vivo	45	WGS, RNA-seq; mIHC	3 iCCA subgroups (sparsely, heterogeneously, and highly infiltrated) correlated to immunogenomics profiles	[226]
Genomics + Transcriptomics + Proteomics	Ex vivo	192	mIHC; Molecular Signatures Database ((MSigDB)	3 iCCA spatial immunophenotypes (inflamed, excluded, and ignored) based on immune infiltration	[227]
Genomics + Transcriptomics	Ex vivo	961	NMF; Transcriptomic tools (i.e., EnrichR); Molecular Signatures Database (MSigDB)	5 iCCA classes (STIM classification) related to the stroma, tumor, and immune microenvironment	[228]
Genomics + Epigenomics + Transcriptomics + Proteomics	Ex vivo	16	WES; RNA-seq; Methylation-Array; mIHC; scRNA-seq; Single-cell RNA-seq	2 iCCA groups according to ITH index (-high and -low)	[229]
Genomics + Transcriptomics + Proteomics	Ex vivo	262	RNA-seq; TCR-seq; FISH; Fusion-derived peptides; peptide-array	4 iCCA subgroups (S1–S4) with specific features in terms of genetic alterations, microenvironment dysregulation, tumor microbiota composition, and OS	[230]
Genomics + Transcriptomics + Proteomics	Ex vivo	102	WES; RNA-seq; LC-MS/MS	3 iCCA clinical subtypes (stem-like, poorly immunogenic, and metabolism)	[231]
Genomics + Transcriptomics + Proteomics	Ex vivo	116	WES; RNA-seq; MS	3 iCCA subgroups (S1–S3) according to metabolomics profile	[232]
